# Iron Deficiency and Overload Modulate the Inflammatory Responses and Metabolism of Alveolar Macrophages

**DOI:** 10.3390/nu14153100

**Published:** 2022-07-28

**Authors:** Vivian Perng, Shya E. Navazesh, Jungjae Park, Joseph R. Arballo, Peng Ji

**Affiliations:** Department of Nutrition, University of California Davis, 1 Shields Ave., Davis, CA 95616, USA; vperng@ucdavis.edu (V.P.); senavazesh@ucdavis.edu (S.E.N.); jnjpark@ucdavis.edu (J.P.); jarballo@ucdavis.edu (J.R.A.)

**Keywords:** iron deficiency, iron overload, immunometabolism, itaconic acid, alveolar macrophage

## Abstract

Alveolar macrophages (AM) are critical to defense against respiratory pathogens. This study evaluated cellular iron imbalance to immunometabolism in endotoxin-polarized porcine AMs (PAMs). PAMs collected from five 6-week-old pigs were treated with a basal media, iron chelator, or ferric ammonium citrate to maintain iron replete or induce iron deficiency or overload, respectively. After 24 h treatment, PAMs were challenged with saline or lipopolysaccharide (LPS) for 6 h. Cells were analyzed for gene, protein, and untargeted metabolome. Cytokines were determined in culture media. Data were assessed using two-way ANOVA. Treatments successfully induced iron deficiency and overload. The mRNA of *DMT1* and *ZIP14* was increased up to 300-fold by LPS, but unaffected by iron. Surprisingly, both iron deprivation and overload attenuated LPS-induced inflammation, showing less TNFα production and lower mRNA of pro- and anti-inflammatory cytokines than iron-replete PAMs. Forty-eight metabolites were altered by either or both main effects. LPS enhanced the glycolysis and polyol pathways. Iron deprivation disrupted the TCA cycle. Iron overload increased intracellular cholesterol. Interestingly, iron deprivation augmented, whereas iron overload diminished, LPS-induced itaconic acid production, which has anti-microbial and anti-inflammatory properties. Therefore, iron-deficient PAMs may be more resistant to intracellular pathogens which use PAMs as a conduit for infection.

## 1. Introduction

Iron is pivotal to host–pathogen interactions by modulating both the host defense and pathogenesis of most bacterial pathogens. Disruption of iron homeostasis in a human host affects innate and adaptive immune responses in various ways [1,2]. Macrophages are critical to host defense and play a key role in regulating systemic iron homeostasis in health and diseases [3]. In the lower respiratory tract, alveolar macrophages (AMs) constitute the major cell population in the niche of the alveolar interstitial space. An approximate 80% of iron detected in the alveolar and interstitial region is present in AMs, suggesting its important role in regulating local iron homeostasis [4]. Alveolar macrophages can acquire both transferrin-bound irons released by alveolar epithelial cells and inhaled iron from environmental exposure (e.g., smoke and air pollution) [5]. In a study with a rat model, dietary iron depletion increased, whereas a high-iron diet decreased the mRNA expression of transferrin receptor 1 and ferroportin in the AMs, suggesting that the cellular iron flux of AMs is affected by the systemic iron balance of the host [6]. Evidence from clinical trials supports an association between host iron status and the risk of lower respiratory tract infections [7,8,9]. Although AMs serve as the first line of defense against inhaled pathogens and involve tissue repair, limited research has evaluated iron deficiency and overload to the immune robustness and iron handling of AMs following pathogen exposure [6]. Ward et al. (2009) reported that AMs isolated from iron-deficient rats displayed attenuated NF-κB activation by LPS compared with AMs from a control group [6]. The regulation of iron trafficking also implicates the pulmonary immune response. This is supported by the fact that the loss of DMT1, an iron transporter, exacerbated LPS-induced pulmonary inflammation [10]. Unfortunately, polarization features of AM were not characterized in this study.

Altered iron availability affects the survival of bacterial pathogens such as Mycobacterium tuberculosis, which infects and replicates in AMs, and whose growth is likely repressed in a low-iron environment and promoted in iron-loading AMs [11]. In addition to the direct effect on pathogenesis, cellular iron homeostasis was shown to modulate macrophage polarization and the production of inflammation effectors [12,13,14]. Iron overloading of macrophages observed in human chronic venous leg ulcers and a mouse model resulted in more profound M1 polarization and lasting inflammation in vivo [15]. Conversely, acute iron deprivation in human macrophages dampened LPS-triggered pro-inflammatory cytokine production including IL-1β and TNFα [14]. However, conflicting results were reported by other studies, wherein spleen macrophages from iron-deficient mice had lower mRNA expression of pro-inflammatory cytokines compared with the control, whereas the iron loading of peritoneal macrophages attenuated M1 activation by LPS [12,13]. Macrophages collected from different sites may handle iron imbalance differently during an immune challenge, therefore causing the varied results among studies. The immunomodulatory effects of iron have been recently linked to its role in intermediary metabolism and cellular energetics [14]. In a study mentioned above, acute treatment of human macrophages with iron chelator shifted energy production from oxidative phosphorylation to glycolysis. This was correlated with decreased aconitase activity and succinate dehydrogenase complex subunit B protein, both of which are iron-requiring enzymes [14]. Furthermore, when stimulated by LPS, the iron-deficient macrophages had a greater intracellular accumulation of itaconate, a metabolite which displays immunosuppressant property, possibly explaining the diminished M1 activation [14,16]. Using a porcine alveolar macrophage (PAM), the current study aimed to understand the immunometabolic response of PAM to bacterial endotoxin, LPS, in the context of iron deficiency and iron overload.

## 2. Materials and Methods

### 2.1. Alveolar Macrophage Collection and Treatment

Alveolar macrophage cells were harvested by bronchoalveolar lavage (BAL) of the lungs extracted from 5 healthy weanling piglets after euthanasia. The trachea and lung were removed immediately after euthanasia. Precautions were taken to avoid blood flow into the trachea. A transfer pipette was inserted into the trachea towards the lung and used to infuse 50–80 mL of ice-cold sterile PBS solution. The lung was gently massaged, and lavage fluid was recovered by filtering through sterile gauze pads. The lavage fluid was centrifuged at 400× *g* at 4 °C for 5 min. The supernatant was removed, and the cell pellet was resuspended in RPMI 1640 supplemented with 10% fetal bovine serum (Gibco, Waltham , MA, USA) and 1% penicillin–streptomycin (50 U/mL). Cells were counted using a hemocytometer and determined for viability through the trypan blue exclusion test. Cells were seeded in culture plates and incubated to adherence at 37 °C with 5% CO_2_. Cell culture was washed with RPMI 1640 to remove nonattached cells before applying treatments. The experiment was designed as a 3 (cellular iron statuses) × 2 (with or without LPS challenge) factorial arrangement of treatments. Cells were treated for 24 h with the complete culture medium as iron adequacy (CON) or the complete culture medium containing either 500 µM deferiprone (Apexbio Technology, Boston, MA, USA) for iron deprivation (DFE) or 200 µM ferric ammonium citrate as iron excess (FAC). At the end of the 24 h treatments, cells remained in the treatment media and were challenged with 100 ng/mL LPS (L, LPS from *E. coli* O111: B4, Sigma-Aldrich, St. Louis, MO, USA) or sterile PBS solution (S) for another 6 h, after which cells or culture media were processed and used in different assays. 

### 2.2. Cell Viability Assay

Cell viability was determined at the end of the LPS challenge using an XTT cell viability assay kit (Biotium, Fremont, CA, USA) following the manufacturer’s instructions. Four technical replicates were tested for each biological sample.

### 2.3. Reverse Transcription-Quantitative PCR (RT-qPCR)

Total RNA was extracted from PAMs using the TRIzol reagent (Invitrogen, Waltham, MA, USA), and cDNA was synthesized from 1 µg of total RNA using the High-Capacity cDNA Reverse Transcription Kit with RNase inhibitor (Applied Biosystems, Waltham, MA, USA). The real-time quantitative PCR was performed in QuantStudio 3 (Applied Biosystems, Waltham, MA, USA) using PowerUp SYBR Green Master Mix (Applied Biosystems, Waltham, MA, USA). The relative gene expression was calculated via the comparative Ct method, with each sample being normalized to the Ct value of the housekeeping gene (Rn18s) and compared with the average ΔCt value of the CON samples. Sequences of primer pairs are provided in Table S1.

### 2.4. Western Blot

The PAMs were lysed in RIPA lysis buffer containing a protease inhibitor cocktail (Sigma-Aldrich, St. Louis, MO, USA). Total protein was harvested and analyzed for concentration via a Micro BCA Protein Assay Kit (Thermo Fisher Scientific, Waltham, MA, USA). The protein sample (30 µg) was loaded for electrophoresis and transferred to a nitrocellulose membrane. The membrane was immunoblotted against the primary antibody H-ferritin (ab231253; Abcam, Cambridge, UK; at 1:1000) and the fluorophore-conjugated secondary antibody (Jackson ImmunoResearch Laboratories; anti-rabbit Alexa680; at 1:3000). The band density of the target protein was normalized to the total protein detected using the ChemiDoc MP imaging system (Bio-Rad, Hercules, CA, USA). The relative expression of H-ferritin was quantified by comparing it to the average density of the CON samples using Image Lab software.

### 2.5. ELISA Assay

Concentrations of TNFα, IL-6, and IL-10 in cell culture media were determined through porcine-specific ELISA assay kits (R&D Systems, Minneapolis, MN, USA) following the manufacturer’s instructions. The absorbance was measured using a Synergy HTX microplate reader (BioTek, Santa Clara, CA, USA).

### 2.6. Untargeted Metabolomics

The growth media were removed from cell culture plates at the end of the experiment. Cells were gently washed with PBS and, subsequently, 1 mL ice-cold 80% methanol (extraction solvent) was added to extract metabolites. Cells were scraped off the plate with the extraction solvent, sonicated in an ice bath for 30 s, and centrifuged at 20,000× *g* at 4 °C for 15 min to harvest supernatants. Supernatants were sent to the West Coast Metabolomics Center for untargeted metabolome analysis using a protocol that was previously described [17].

### 2.7. Statistical Analysis

Cells collected from each donor pig were considered a biological replicate. Data were assessed through two-way ANOVA using the MIXED procedure of SAS (v. 9.4). Data were tested for compliance with the two assumptions (normal distribution and homogeneity of variance) using UNIVARIATE and GLM procedures of SAS, respectively. Data that violated assumptions were subjected to logarithmic transformation. The results presented were based on original data (non-transformed). Statistical significance was declared at *p* ≤ 0.05. Metabolomics data are shown as peak intensities and were normalized by natural log transformation and autoscaling. Normalized data were assessed through univariate two-way ANOVA (ANOVA2) using the MetaboAnalyst platform (v. 4.0). Statistical significance was declared at FDR adjusted *p* < 0.1. A heatmap of altered metabolites (FDR < 0.1) was created based on the relative fold change compared with the mean peak intensity of the metabolite of CON-S samples.

## 3. Results

### 3.1. Cytotoxicity of LPS

**Figure 1:**
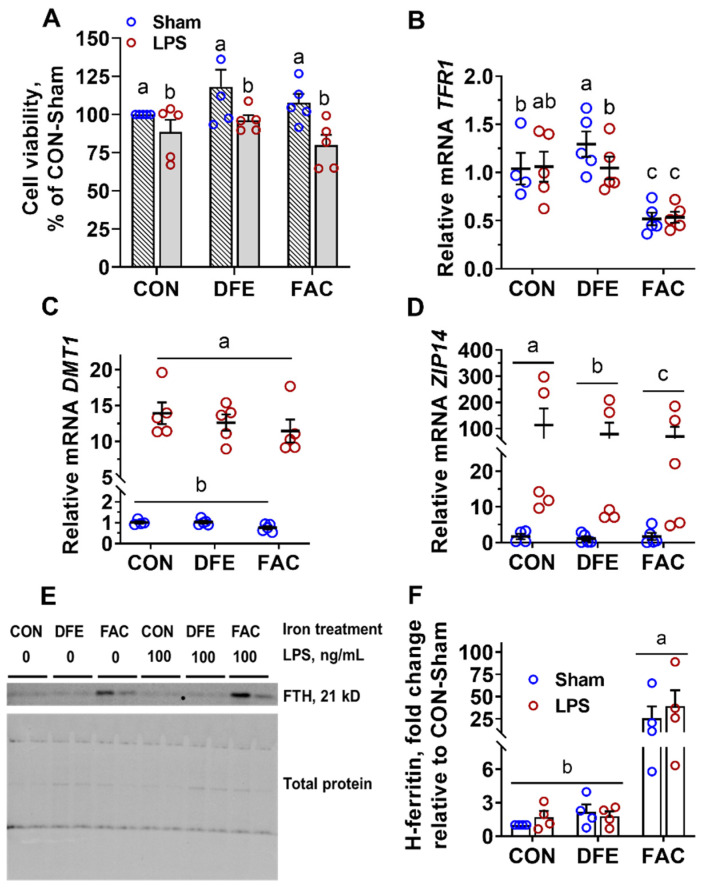
Effects of LPS (100 ng/mL, 6 h) challenge on cell viability and iron metabolism of PAMs pre-exposed to deferiprone (DFE, 500 µM, 24 h) or ferric ammonium citrate (FAC, 200 µM, 24 h). (**A**) LPS reduced cell viability independent of cellular iron status (*P*_Fe_ = 0.11, *P*_LPS_ = 0.001, *P*_Fe×LPS_ = 0.49). (**B**) Iron deprivation increased, whereas iron excess decreased, *TFR1* mRNA expression (*P*_Fe_ < 0.001, *P*_LPS_ = 0.15, *P*_Fe×LPS_ = 0.04). (**C**) LPS markedly increased *DMT1* mRNA expression (*P*_Fe_ = 0.07, *P*_LPS_ < 0.001, *P*_Fe×LPS_ = 0.17). (**D**) Iron imbalance decreased and LPS increased *ZIP14* mRNA expression (*P*_Fe_ = 0.004, *P*_LPS_ < 0.001, *P*_Fe×LPS_ = 0.44). (**E**) Sample image of H-ferritin (FTH) immunoblot. (**F**) Iron overexposure increased FTH protein expression in PAMs (*P*_Fe_ < 0.001, *P*_LPS_ = 0.42, *P*_Fe×LPS_ = 0.60). Treatments sharing no common letter (a, b, c) are significantly different (*p* < 0.05).

Iron treatment did not alter cell viability (*p* = 0.11, Figure 1A), suggesting that PAMs are tolerant of acute iron chelation and iron overexposure treatments used in the current study. The cytotoxicity of bacterial LPS on various types of cells, including macrophages, is mediated through the stimulation of nitric oxide production (Yamamoto et al., 1994). Accordingly, there was a modest reduction in cell viability in LPS-challenged PAMs, regardless of the cellular iron status in the current study (*p* = 0.001).

### 3.2. Iron Depletion and Overload Attenuated LPS-Induced Inflammatory Response in PAM

The first aim of the study was to understand how iron chelators and iron excess affect the iron handling of PAM following LPS exposure. High iron exposure (FAC) significantly decreased the mRNA expression of TFR1 compared with CON and DFE (*p* < 0.05, Figure 1B). In the absence of LPS challenge, treatment with DFE for 24 h significantly increased *TFR1* expression compared with CON (*p* < 0.05), indicative of cellular iron deficiency. Iron excess resulted in a drastic increase in H-ferritin, an iron storage protein, compared with CON and DFE, regardless of the LPS stimulation (*p* < 0.001, Figure 1E,F and Figure S1), suggesting cellular iron overload. Neither iron depletion nor overload affected the mRNA expression of *DMT1* (*p* = 0.07, Figure 1C), although both types of iron imbalance decreased *ZIP14* compared with CON (*p* = 0.004, Figure 1D). Interestingly, exposure to LPS markedly increased the expression of both metal transporters (*DMT1* and *ZIP14*, *p* < 0.001). The study showed that changes in iron availability primarily affect *TFR1* and ferritin expression, whereas the LPS challenge had a major impact on the expression of *DMT1* and *ZIP14* in PAMs.

**Figure 2:**
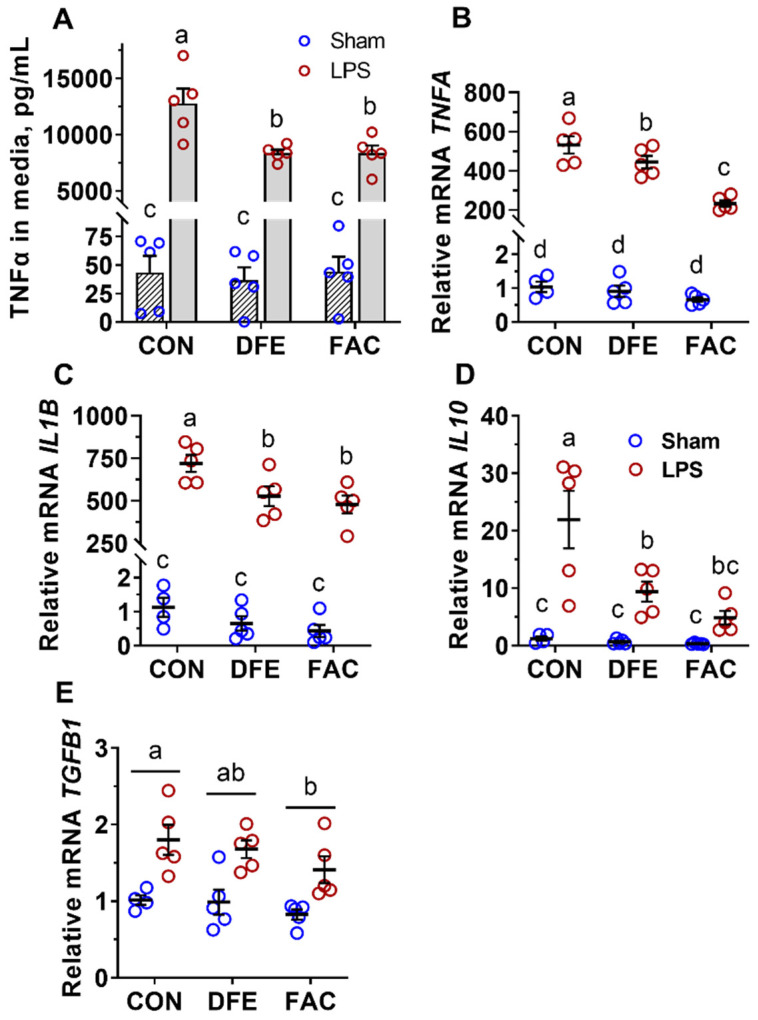
Effects of LPS (100 ng/mL, 6 h) challenge on the inflammatory response of PAMs pre-exposed to deferiprone (DFE, 500 µM, 24 h) or ferric ammonium citrate (FAC, 200 µM, 24 h). (**A**) Iron imbalance attenuated TNFα production from LPS-polarized PAMs (*P*_Fe_ < 0.001, *P*_LPS_ < 0.001, *P*_Fe×LPS_ < 0.001). (**B**–**D**) Both types of iron imbalance decreased mRNA expression of TNFA, IL1B and IL10 in LPS-polarized PAMs (*P*_Fe_ < 0.001, *P*_LPS_ < 0.001, *P*_Fe×LPS_ < 0.001). (**E**) LPS increased and iron overload decreased the mRNA expression of TGFB1 in PAMs compared with the control (CON) or non-stimulated groups (*P*_Fe_ = 0.014, *P*_LPS_ < 0.001, *P*_Fe×LPS_ = 0.60). Treatments sharing no common letter (a, b, c) are significantly different (*p* < 0.05).

As expected, the LPS challenge sharply increased the TNFα concentration in culture media (*p* < 0.001, Figure 2A). However, it is interesting that both iron deprivation and cellular iron overload dampened LPS-stimulated TNFα production (*p* < 0.001). Similarly, DFE and FAC treatment attenuated the LPS-induced expression of pro-inflammatory (*TNFA* and *IL1B*) and anti-inflammatory (*TGFB* and *IL10*) cytokines in PAMs (*p* ≤ 0.014, Figure 2B–E).

### 3.3. Iron Imbalance and LPS Challenge Modulated the Metabolism of PAM

**Figure 3:**
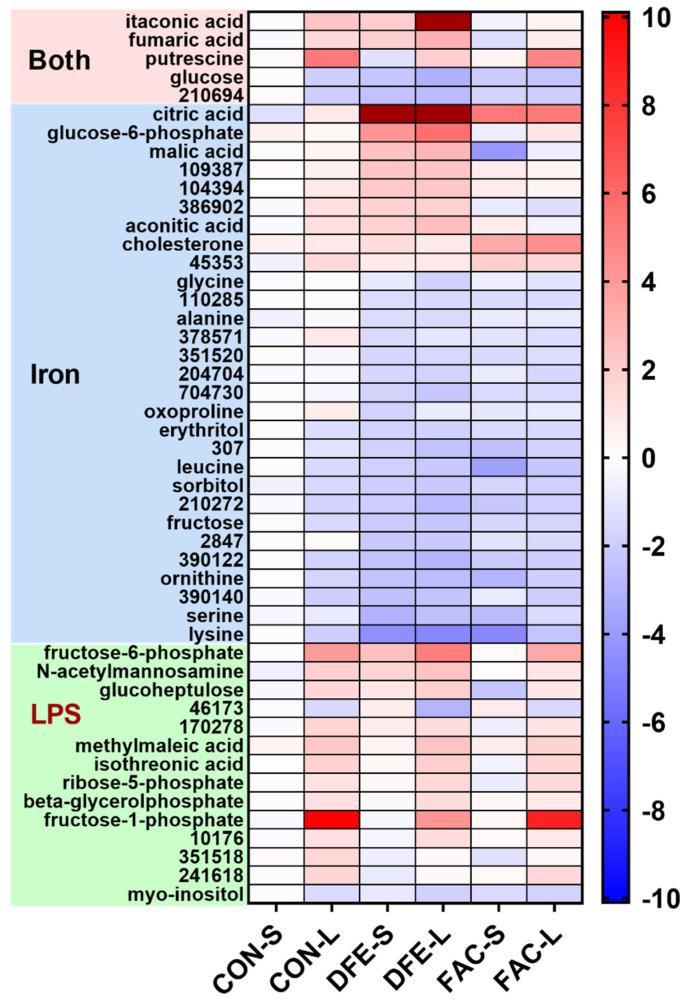
Untargeted metabolomics revealed 48 metabolites (28 identified) of PAMs that were altered by LPS (100 ng/mL, 6 h) challenge or pre-challenge exposure to deferiprone (DFE, 500 µM, 24 h) or ferric ammonium citrate (FAC, 200 µM, 24 h) or both effects. Results are presented as the fold change relative to the non-challenged control group (CON-S). CON-S, DFE-S and FAC-S: cells were pre-exposed to iron-replete control media, DFE-containing media, and FAC-containing media, respectively, and challenged with sterile PBS solution. CON-L, DFE-L and FAC-L: cells were pre-exposed to iron-replete control media, DFE-containing media, and FAC-containing media, respectively, and challenged with LPS.

The untargeted metabolomics analysis detected 335 metabolites, of which 113 were identified in the user’s library (Table S2). None of the metabolites were affected by the interaction of iron and LPS (adjusted *p* ≥ 0.10). Iron imbalance had a profound effect on 29 metabolites, among which 15 were identified in the user database (adjusted *p* < 0.10, Figure 3, Table S2). Iron deficiency led to the marked accumulation of citric acid as well as other glycolytic and TCA cycle intermediates, including glucose-6-phosphate, aconitic acid, and malic acid; however, iron overload enhanced cholesterol synthesis. Both types of iron imbalance decreased fructose, erythritol, sorbitol, and several amino acids (glycine, alanine, lysine, ornithine, leucine, and serine) or amino acid derivative (oxoproline). There were 14 metabolites (9 identified) primarily affected by the LPS challenge (adjusted *p* < 0.10). Notably, key metabolites involved in the pentose phosphate pathway (fructose-1-phosphate, fructose-6-phosphate, and ribose-5-phosphate) were significantly increased by LPS. Myo-inositol was the only identified metabolite that was decreased by LPS. Five metabolites (four identified) were subjected to the modulation of both main effects (adjusted *p* < 0.10). Notably, itaconic acid and fumaric acid were significantly increased by LPS stimulation, no matter the cellular iron status; iron deficiency enhanced, but iron overload tended to decrease both metabolites compared with the CON, regardless of the LPS stimulation. LPS was also a strong inducer for putrescine, whereas iron deficiency decreased, and iron overload did not seem to affect its level. Both LPS and iron imbalance decreased glucose levels; there tended to be a greater reduction in iron-deficient cells. Altogether, the altered metabolites highlighted changes in the metabolic flux of the TCA cycle, glycolysis, and pentose phosphate pathways in response to changes in cellular iron status and LPS challenge.

## 4. Discussion

This study showed clear evidence that the iron metabolism of PAM is subjected to the regulation of environmental iron availability and the immune challenge. The mRNA stability of TFR1 and translation of ferritin genes are regulated by changes in cellular iron via the well-characterized IRP/IRE system [18]. Therefore, the mRNA levels of TFR1 and ferritin protein are surrogate markers for intracellular iron; the highest expression of *TFR1* in DFE-S cells indicated cellular iron depletion, whereas the lowest expression of *TFR1*, along with the highest H-ferritin protein, in FAC-treated PAMs suggested iron overloading. In line with our findings, *TFR1* was upregulated in AMs in iron-deficient rats and downregulated in rats with chronic iron overload [6]. The role of DMT1 in macrophages is not completely understood. *DMT1* expression was not affected by iron imbalance in the current study; therefore, it is unlikely to be involved in extracellular non-heme iron uptake due to its well-defined role in enterocytes, but, instead, contributed to transporting iron among subcellular compartments. Indeed, DMT1 was shown to participate in transporting iron from the phagolysosome to cytosol; defective *DMT1* expression reduced the labile iron pool and impaired iron release in RAW264.7 macrophages [19]. Similar to our finding, others have also observed a striking induction of *DMT1* in macrophages by LPS, suggesting a role in the inflammatory response [20,21]. Belgrade rats with nonfunctional DMT1 exhibited exacerbated pulmonary inflammation following the intratracheal instillation of LPS [10]. Therefore, we postulate that DMT1 regulates the labile iron pool to restrain uncontrolled inflammatory responses in PAMs. *ZIP14* encodes a zinc transporter that could also transport non-transferrin-bound iron [22]. Lipopolysaccharide is a strong inducer of *ZIP14* expression, as observed with PAMs in the current study and with human macrophages, whereas inhibiting *ZIP14* significantly enhanced the mRNAs of IL-6 and TNFα [23]. In addition, high intracellular zinc repressed NF-κB signaling; thus, it is conceivable that the induction of *ZIP14* during PAM polarization by LPS prevents inordinate inflammatory responses. Nevertheless, the expression patterns of *DMT1* and *ZIP14* cannot explain the attenuated inflammation in DFE- and FAC-treated PAMs.

The study further examined the effect of iron imbalance on LPS-induced PAM polarization. Surprisingly, both iron deficiency and overload dampened TNFα production and diminished the mRNA expression of both pro- and anti-inflammatory cytokines in LPS-activated PAMs. Notably, the mRNA levels of *TNFα* (mean Ct = ~16) and *IL1β* (mean Ct = ~14) were much more abundant than those of transforming growth factor-beta 1 (*TGFB1*) (mean Ct = ~21) and *IL10* (mean Ct = ~24) in all treatments (data not shown); in addition, in comparison with the sharply increased TNFα in the culture medium by LPS, the protein concentration of IL-10 was below the lowest detection limit of the ELISA kit (R&D systems; data not shown), suggesting that most PAMs were still undergoing pro-inflammatory activation (M1 type) at 6 h of LPS challenge. Thus, it appeared that neither iron-deficient nor iron-overloaded PAMs mounted as robust an inflammatory response to LPS as iron-replete control cells. Studies that assessed the impact of iron deficiency on macrophage polarization yielded more consistent results. For example, intracellular iron deficiency induced by either iron chelation or overexpression of ferroportin, the only known iron exporter, attenuated LPS-stimulated TNFα and IL-6 production in peritoneal macrophages [24]. In addition, human macrophages treated with an iron chelator (500 µM DFE, 24 h) showed a greater expression of anti-inflammatory genes involved in TGF-β signaling (e.g., *TGFB1*, *IL10*, and *VEGFA*) at 3 h of LPS stimulation (100 ng/mL) and secreted less TNFα and IL-1β after 24 h of LPS exposure, suggesting an early induction of anti-inflammatory signaling by acute iron deprivation [14]. Unfortunately, pro-inflammatory cytokines were not measured at 3 h of LPS treatment, nor were anti-inflammatory genes determined at 24 h of LPS treatment [14]. In the current study, it is unlikely that the attenuated classical activation in DFE- and FAC-treated PAMs is due to a prompt switch to alternative activation (M2 type), because the expression of anti-inflammatory cytokines would otherwise be higher compared with iron-replete PAMs. Studies have reported conflicting results regarding the impact of intracellular iron overload on macrophage polarization. Gan et al. (2017) found that pretreatment with high iron levels (FAC, 25 µg/mL) significantly reduced the mRNA expression of pro-inflammatory cytokines and iNOS production, while enhancing phagocytic activity in IFN-ɣ-polarized RAW264.7 macrophages [25]. In contrast, iron overload was shown to promote classical activation (M1 type), with or without the presence of stimuli, and amplify inflammation in various settings in vivo or in vitro [12,26,27]. Although it is still an enigma, the effect of iron overload on macrophage polarization is likely dependent on the experimental setting.

It has increasingly been recognized that metabolic reprogramming during macrophage polarization is not simply an adaptive response, but also methodically tunes innate immune activity [28,29]. In the current study, the LPS challenge significantly decreased cellular glucose, while glucose-6-phosphate (numerically) and fructose-6-phosphate were increased, which is seemingly indicative of enhanced glycolysis. Others have also observed that LPS-stimulated macrophages and dendritic cells were associated with the upregulation of glycolysis, a pathway of rapid energy production which is believed to compensate for impaired TCA cycle and mitochondrial respiration during inflammatory activation [28,30]. Intriguingly, metabolites of the polyol pathway, including sorbitol, fructose, and fructose-1-phosphate, were altered by LPS stimulation in CON PAMs or all PAMs, presumably indicating the activation of an additional route to catabolize cellular glucose during pro-inflammatory activation. This is in agreement with the finding that aldose reductase, the rate-limiting enzyme of the polyol pathway, was upregulated and mediated inflammatory signals in M1-polarized macrophages [31,32]. It is unclear why the metabolic flux of glycolysis and the polyol pathway are built up at fructose-6-phosphate and fructose-1-phosphate, respectively. It is possibly due to the feedback inhibition by downstream products (e.g., citrate). Myo-inositol is synthesized from glucose-6-phosphate; reductions in myo-inositol by LPS likely result from sparing glucose for glycolysis in activated PAMs. Itaconic acid is produced from cis-aconitate by aconitate decarboxylase (ACOD1) in immune cells. The induction of ACOD1 expression and intracellular accumulation of itaconic acid are hallmarks of the pro-inflammatory activation of macrophages [33,34]. Consistently, itaconic acid was significantly higher in LPS-stimulated PAMs in the current study. Putrescine is a diamine produced from the decarboxylation of ornithine, a reaction catalyzed by ornithine decarboxylase (ODC) in mammalian cells [35]. The enzyme activity of ODC was markedly increased in peritoneal macrophages at 6 to 9 h of LPS exposure in vitro [36]. Thus, our finding of increased putrescine by LPS was likely due to the induction of ODC. Interestingly, putrescine seems to have anti-inflammatory properties, because the loss of ODC exacerbated the inflammatory response of bone-marrow-derived macrophages to *H. pylori* in vitro, which was restored with the addition of putrescine in cell culture [37]. However, this notion does not explain the diminished inflammatory profile observed in iron-imbalanced PAMs, which should exhibit higher putrescine compared with the CON PAMs otherwise.

Studies have suggested that iron deficiency is associated with the altered risk of lower respiratory tract infections, but the role of AMs in host defense under iron deficiency is rarely investigated [7,8,9]. Intracellular iron homeostasis of AM is subject to modulation by diseases and environmental exposure [38,39]. Given the essential role of iron in cellular metabolism, the metabolic profiles of PAMs associated with iron deficiency and overload may be causally linked to the altered inflammatory response to LPS. In the current study, the most notable finding is that, compared with the CON groups, iron deprivation enhanced, while iron overload decreased, the cellular accumulation of itaconic acid in both non-stimulated and LPS-stimulated PAMs. The implications of this finding are manifold. Firstly, itaconate displays the antimicrobial property that is likely mediated through inhibiting bacterial isocitrate lyase, a key enzyme in the glyoxylate cycle that regulates microbial growth and survival [33,40]. Host iron status was reported to be causally linked to the susceptibility to intracellular bacterial pathogens (e.g., *Mycobacterium tuberculosis*) that use alveolar macrophages as the primary conduit of infection [7,41]. Hence, iron-induced changes in intracellular itaconate in PAMs may serve as a previously underappreciated cellular mechanism in the defense against intracellular pathogens. Moreover, itaconic acid demonstrates an anti-inflammatory effect at least by inhibiting SDH activity, while activating antioxidant transcription factor Nrf2 [29,42]. In the current study, the diminished pro-inflammatory profile of LPS-polarized iron-deficient PAMs, but not iron-overloaded PAMs, could therefore be ascribed to the augmented production of itaconic acid. It is conceivable that iron deprivation compromised the activity of aconitase, an iron-dependent enzyme, and thus shifted aconitate from the TCA cycle toward the synthesis of itaconic acid by ACOD1 in both non- and LPS-stimulated PAMs. This is further supported by the marked accumulation of glucose-derived citric acid and a moderate increase in aconitic acid in iron-deficient PAMs. Our results are in line with another study wherein acute iron deprivation compromised oxidative phosphorylation, led to the cellular accumulation of citrate, itaconate, and aconitate in human bone-marrow-derived macrophages, and dampened pro-inflammatory cytokine production after the LPS challenge [14]. Impaired oxidative phosphorylation by iron deficiency has long been attributed to diminished aconitase activity and truncated TCA reactions; however, the study by Lampropoulou et al. (2016) showed that the induction of itaconate also compromised mitochondria respiration by inhibiting the activity of succinate dehydrogenase (SDH), an enzyme complex which contains Fe–S clusters and contributes to both the TCA cycle and mitochondrial electron transport [42]. The accumulation of fumaric acid and malic acid in iron-deficient PAMs also suggested a disrupted TCA cycle compared with iron-replete cells. Glucose-6-phosphate was also the highest level in iron-deficient PAMs, suggesting increased dependence on glycolysis for energy production. The accumulation of TCA intermediates discussed above was not observed in iron-overloaded PAMs, except for citric acid. Ferric ammonium citrate was used as the iron supplement in the IE treatment; thus, it is plausible that greater citric acid in iron-overloaded PAMs was derived from culture media instead of glycolysis. Altogether, iron overloading did not seem to interrupt the TCA cycle, but increased intracellular cholesterol in non-activated and M1 polarized PAMs. Cai et al. (2020) reported that iron deposition in macrophages downregulated the expression of ABC transporters and thus reduced the cholesterol efflux [43]. Lastly, given the lowest itaconic acid in FAC-treated PAMs, the possible mechanism contributing to the moderate inflammatory response in iron-overloaded PAMs was unknown in the current study.

## 5. Conclusions

In the current study, both iron deficiency and overload attenuated the LPS-stimulated inflammatory response in PAMs. Metabolite profiling suggested altered glycolysis and polyol pathways and the production of itaconic acid to be the most prominent metabolic signatures in LPS-polarized PAMs. Deferiprone-induced cellular iron deficiency may have disrupted the TCA cycle and potentiated LPS-stimulated itaconic acid production, which may contribute to the diminished inflammatory response. Iron overload suppressed LPS-stimulated itaconic acid production while increasing intracellular cholesterol. Mechanisms leading to the less robust inflammatory response in iron-overloaded PAMs are enigmatic and cannot be attributed to changes in metabolomics profiles. The current study is limited by the single time point of sample collection. A time–course study of metabolomics is warranted in future research.

## Data Availability

Not applicable.

## Supplementary Materials

The following supporting information can be downloaded at: https://www.mdpi.com/article/10.3390/nu14153100/s1, Figure S1: Additional sample image of H-ferritin (FTH) immunoblot; Table S1: Sequences of primers for tested genes; Table S2: Statistical results for all detected metabolites.

## References

[1] Cassat J.E., Skaar E.P. (2013). Iron in infection and immunity. Cell Host Microbe.

[2] Weiss G. (2002). Iron and immunity: A double-edged sword. Eur. J. Clin. Investig..

[3] Ganz T. (2012). Macrophages and systemic iron homeostasis. J. Innate Immun..

[4] Corhay J.L., Weber G., Bury T., Mariz S., Roelandts I., Radermecker M.F. (1992). Iron content in human alveolar macrophages. Eur. Respir. J..

[5] Holian A., Scheule R.K. (1990). Alveolar macrophage biology. Hosp. Pract..

[6] Ward R.J., Wilmet S., Legssyer R., Leroy D., Toussaint L., Crichton R.R., Pierreux C., Hue L., Piette J., Srai S.K. (2009). Effects of marginal iron overload on iron homeostasis and immune function in alveolar macrophages isolated from pregnant and normal rats. Biometals.

[7] Gangaidzo I.T., Moyo V.M., Mvundura E., Aggrey G., Murphree N.L., Khumalo H., Saungweme T., Kasvosve I., Gomo Z.A., Rouault T. (2001). Association of pulmonary tuberculosis with increased dietary iron. J. Infect. Dis..

[8] Stepan D., Dop D., Morosanu A., Vintilescu B., Niculescu C. (2018). Implications of the iron deficiency in lower tract respiratory acute infections in toddlers. Curr. Health Sci. J..

[9] Wander K., Shell-Duncan B., Brindle E. (2017). Lower incidence of respiratory infections among iron-deficient children in kilimanjaro, tanzania. Evol. Med. Public Health.

[10] Kim J., Molina R.M., Donaghey T.C., Buckett P.D., Brain J.D., Wessling-Resnick M. (2011). Influence of dmt1 and iron status on inflammatory responses in the lung. Am. J. Physiol. Lung Cell Mol. Physiol..

[11] Boelaert J.R., Vandecasteele S.J., Appelberg R., Gordeuk V.R. (2007). The effect of the host’s iron status on tuberculosis. J. Infect. Dis..

[12] Agoro R., Taleb M., Quesniaux V.F.J., Mura C. (2018). Cell iron status influences macrophage polarization. PLoS ONE.

[13] Pagani A., Nai A., Corna G., Bosurgi L., Rovere-Querini P., Camaschella C., Silvestri L. (2011). Low hepcidin accounts for the proinflammatory status associated with iron deficiency. Blood.

[14] Pereira M., Chen T.D., Buang N., Olona A., Ko J.H., Prendecki M., Costa A.S.H., Nikitopoulou E., Tronci L., Pusey C.D. (2019). Acute iron deprivation reprograms human macrophage metabolism and reduces inflammation in vivo. Cell Rep..

[15] Sindrilaru A., Peters T., Wieschalka S., Baican C., Baican A., Peter H., Hainzl A., Schatz S., Qi Y., Schlecht A. (2011). An unrestrained proinflammatory m1 macrophage population induced by iron impairs wound healing in humans and mice. J. Clin. Investig..

[16] O’Neill L.A.J., Artyomov M.N. (2019). Itaconate: The poster child of metabolic reprogramming in macrophage function. Nat. Rev. Immunol..

[17] Fiehn O. (2016). Metabolomics by gas chromatography-mass spectrometry: Combined targeted and untargeted profiling. Curr. Protoc. Mol. Biol..

[18] Anderson C.P., Shen M., Eisenstein R.S., Leibold E.A. (2012). Mammalian iron metabolism and its control by iron regulatory proteins. Biochim. Biophys. Acta.

[19] Soe-Lin S., Apte S.S., Mikhael M.R., Kayembe L.K., Nie G., Ponka P. (2010). Both nramp1 and dmt1 are necessary for efficient macrophage iron recycling. Exp. Hematol..

[20] Ludwiczek S., Aigner E., Theurl I., Weiss G. (2003). Cytokine-mediated regulation of iron transport in human monocytic cells. Blood.

[21] Wardrop S.L., Richardson D.R. (2000). Interferon-gamma and lipopolysaccharide regulate the expression of nramp2 and increase the uptake of iron from low relative molecular mass complexes by macrophages. Eur. J. Biochem..

[22] Liuzzi J.P., Aydemir F., Nam H., Knutson M.D., Cousins R.J. (2006). Zip14 (Slc39a14) mediates non-transferrin-bound iron uptake into cells. Proc. Natl. Acad. Sci. USA.

[23] Sayadi A., Nguyen A.T., Bard F.A., Bard-Chapeau E.A. (2013). Zip14 expression induced by lipopolysaccharides in macrophages attenuates inflammatory response. Inflamm. Res..

[24] Wang L., Johnson E.E., Shi H.N., Walker W.A., Wessling-Resnick M., Cherayil B.J. (2008). Attenuated inflammatory responses in hemochromatosis reveal a role for iron in the regulation of macrophage cytokine translation. J. Immunol..

[25] Gan Z.S., Wang Q.Q., Li J.H., Wang X.L., Wang Y.Z., Du H.H. (2017). Iron reduces m1 macrophage polarization in raw264.7 macrophages associated with inhibition of STAT1. Mediators Inflamm..

[26] Kroner A., Greenhalgh A.D., Zarruk J.G., Passos Dos Santos R., Gaestel M., David S. (2014). Tnf and increased intracellular iron alter macrophage polarization to a detrimental m1 phenotype in the injured spinal cord. Neuron.

[27] Zhou Y., Que K.T., Zhang Z., Yi Z.J., Zhao P.X., You Y., Gong J.P., Liu Z.J. (2018). Iron overloaded polarizes macrophage to proinflammation phenotype through ros/acetyl-p53 pathway. Cancer Med..

[28] Mills E.L., Kelly B., Logan A., Costa A.S.H., Varma M., Bryant C.E., Tourlomousis P., Dabritz J.H.M., Gottlieb E., Latorre I. (2016). Succinate dehydrogenase supports metabolic repurposing of mitochondria to drive inflammatory macrophages. Cell.

[29] Mills E.L., Ryan D.G., Prag H.A., Dikovskaya D., Menon D., Zaslona Z., Jedrychowski M.P., Costa A.S.H., Higgins M., Hams E. (2018). Itaconate is an anti-inflammatory metabolite that activates nrf2 via alkylation of keap1. Nature.

[30] Jha A.K., Huang S.C., Sergushichev A., Lampropoulou V., Ivanova Y., Loginicheva E., Chmielewski K., Stewart K.M., Ashall J., Everts B. (2015). Network integration of parallel metabolic and transcriptional data reveals metabolic modules that regulate macrophage polarization. Immunity.

[31] Erbel C., Rupp G., Domschke G., Linden F., Akhavanpoor M., Doesch A.O., Katus H.A., Gleissner C.A. (2016). Differential regulation of aldose reductase expression during macrophage polarization depends on hyperglycemia. Innate Immun..

[32] Ramana K.V., Srivastava S.K. (2006). Mediation of aldose reductase in lipopolysaccharide-induced inflammatory signals in mouse peritoneal macrophages. Cytokine.

[33] Michelucci A., Cordes T., Ghelfi J., Pailot A., Reiling N., Goldmann O., Binz T., Wegner A., Tallam A., Rausell A. (2013). Immune-responsive gene 1 protein links metabolism to immunity by catalyzing itaconic acid production. Proc. Natl. Acad. Sci. USA.

[34] Strelko C.L., Lu W., Dufort F.J., Seyfried T.N., Chiles T.C., Rabinowitz J.D., Roberts M.F. (2011). Itaconic acid is a mammalian metabolite induced during macrophage activation. J. Am. Chem. Soc..

[35] Heby O., Persson L. (1990). Molecular genetics of polyamine synthesis in eukaryotic cells. Trends Biochem. Sci..

[36] Prosser F.H., Wahl L.M. (1988). Involvement of the ornithine decarboxylase pathway in macrophage collagenase production. Arch. Biochem. Biophys..

[37] Hardbower D.M., Asim M., Luis P.B., Singh K., Barry D.P., Yang C., Steeves M.A., Cleveland J.L., Schneider C., Piazuelo M.B. (2017). Ornithine decarboxylase regulates m1 macrophage activation and mucosal inflammation via histone modifications. Proc. Natl. Acad. Sci. USA.

[38] Ghio A.J., Soukup J.M., Stonehuerner J., Tong H., Richards J., Gilmour M.I., Madden M.C., Shen Z., Kantrow S.P. (2019). Quartz disrupts iron homeostasis in alveolar macrophages to impact a pro-inflammatory effect. Chem. Res. Toxicol..

[39] Philippot Q., Deslee G., Adair-Kirk T.L., Woods J.C., Byers D., Conradi S., Dury S., Perotin J.M., Lebargy F., Cassan C. (2014). Increased iron sequestration in alveolar macrophages in chronic obstructive pulmonary disease. PLoS ONE.

[40] Rittenhouse J.W., McFadden B.A. (1974). Inhibition of isocitrate lyase from pseudomonas indigofera by itaconate. Arch. Biochem. Biophys..

[41] Schaible U.E., Kaufmann S.H. (2004). Iron and microbial infection. Nat. Rev. Microbiol..

[42] Lampropoulou V., Sergushichev A., Bambouskova M., Nair S., Vincent E.E., Loginicheva E., Cervantes-Barragan L., Ma X., Huang S.C., Griss T. (2016). Itaconate links inhibition of succinate dehydrogenase with macrophage metabolic remodeling and regulation of inflammation. Cell Metab..

[43] Cai J., Zhang M., Liu Y., Li H., Shang L., Xu T., Chen Z., Wang F., Qiao T., Li K. (2020). Iron accumulation in macrophages promotes the formation of foam cells and development of atherosclerosis. Cell Biosci..

