# Supplementary material for: Iron Deficiency and Overload Modulate the Inflammatory Responses and Metabolism of Alveolar Macrophages
Source: Nutrients. 2022 Jul 28;14(15):3100. doi: 10.3390/nu14153100 (PMC9370601; doi:10.3390/nu14153100)
Supplement: Supplementary file 1 [file nutrients-14-03100-s001.zip › nutrients-1760488-supplementary.pdf]

Table S1. Sequences of primers for tested genes.

Gene symbol	Gene name	Forward primer 5′-3′	Reverse primer 5′-3′
TFR1	Transferrin receptor 1	GTTTAGCGCAGGAGTGAGGC	TGGGACAAGCAACAGAGGAA
DMT1 (SLC11A2)	Solute carrier family 11 member 2	AGGATCTAGGGCATGTGGTG	CCACAGTCCAGGAAGGACAT
ZIP14 (SLC39A14)	Solute carrier family 39 member 14	CGAGGAGAACAAGAAGACGG	ACTCACCCTTCCTGGTGATG
TNFα	Tumor necrosis factor	CGTGAAGCTGAAAGACAACCAG	GATGGTGTGAGTGAGGAAAAC
IL1B	Interleukin 1 beta	CCTTGAAACGTGCAATGATG	TTCAAGTCCCCTGTGAGGAG
IL10	Interleukin 10	TCGGCCCAGTGAAGAGTTTC	GGAGTTCACGTGCTCCTTGA
TGFB1	Transforming growth factor beta 1	TCCAAGGACCCTTCTCGGAT	AAAAACCGAGATGGCGAGA
RN18S	18S ribosomal RNA	AGGAAAGCAGACATCGACCT	ACCTGGCTGTACTTCCCATC

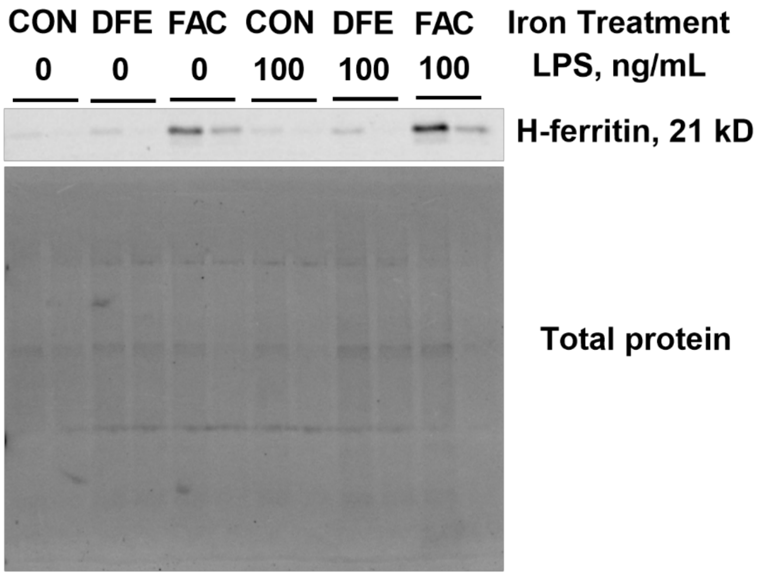

Figure S1. Additional sample image of H-ferritin (FTH) immunoblot.

Table S2. Statistical results for all detected metabolites. Metabolites are reported as peak intensity (mean and standard deviation, SD). Statistical outcomes are presented as raw P-value (raw.p) and FDR adjusted P-value (adj.p) for main effects of iron and LPS and their interaction.

	CON				DFE				FAC				Main Effects				Interaction	
	LPS		Sham		LPS		Sham		LPS		Sham		Iron		LPS			
Metabolites	mean	SD	mean	SD	mean	SD	mean	SD	mean	SD	mean	SD	raw.p	adj.p	raw.p	adj.p	raw.p	adj.p
xylose	5385	2900	6282	3712	2677	1346	7906	10460	6023	4566	5806	3073	0.4082	0.6374	0.2560	0.6937	0.5350	0.9527
xylonic acid isomer	1108	205	1019	281	917	88	940	130	1153	272	984	63	0.2469	0.5076	0.3006	0.7350	0.6202	0.9527
xylitol	1096	296	1133	361	858	215	999	130	1080	296	1135	286	0.3064	0.5519	0.4075	0.8078	0.8152	0.9527
vanillic acid	532	137	541	205	429	339	790	740	821	445	515	226	0.6952	0.8528	0.8086	0.9338	0.1549	0.9527
valine	21151	6436	22440	7827	14747	6883	18761	7764	18220	9360	24850	10153	0.4503	0.6683	0.2546	0.6937	0.7777	0.9527
uracil	1464	540	1210	530	1442	604	937	505	1174	600	1257	565	0.7706	0.8695	0.2575	0.6937	0.4785	0.9527
UDP GlcNAc	627	100	544	219	841	273	988	255	621	212	705	167	0.0118	0.1077	0.6794	0.9169	0.3523	0.9527
tyrosine	18760	6434	29163	13032	12778	2545	13678	6989	16910	4529	13037	6616	0.0196	0.1436	0.9974	0.9974	0.1619	0.9527
tryptophan	941	443	2230	2064	616	202	1149	1052	1261	589	832	304	0.2850	0.5304	0.3682	0.7855	0.1764	0.9527
triethanolamine	630	108	593	155	763	302	1265	1304	700	227	772	255	0.2717	0.5230	0.5212	0.8338	0.6444	0.9527
trans-4-hydroxyproline	25960	9161	19967	6443	20118	8292	12058	5823	19248	8267	15819	7583	0.0925	0.3162	0.0418	0.2745	0.6274	0.9527
threonine	9206	2180	11331	3749	4952	1046	5391	1710	8072	1172	6270	3261	0.0193	0.1436	0.6057	0.8664	0.2533	0.9527
threonic acid	753	230	631	170	598	244	760	475	753	434	719	148	0.8385	0.9150	0.8353	0.9422	0.6793	0.9527
sucrose	894	375	895	221	856	473	1877	1774	1337	721	1345	765	0.5264	0.7169	0.3553	0.7837	0.6438	0.9527
succinic acid	7266	982	7892	595	8116	3503	7172	2908	6787	831	6257	1620	0.3634	0.6027	0.6118	0.8664	0.5940	0.9527
spermidine	1799	845	3520	4867	761	188	830	359	1521	339	2393	3579	0.0871	0.3162	0.8676	0.9452	0.7125	0.9527
sorbitol	35625	8532	54748	7615	26144	4526	28317	6456	30906	8547	34258	8334	0.0002	0.0044	0.0164	0.1768	0.1323	0.9527
serotonin	1263	266	1162	607	936	235	954	366	819	173	753	253	0.0304	0.1710	0.4289	0.8243	0.9043	0.9648
serine	13526	3060	15848	9290	6636	1788	5910	2481	11873	3021	9129	4461	0.0022	0.0283	0.3206	0.7565	0.5645	0.9527
salicylic acid	1305	457	985	302	1053	364	962	117	1119	392	1074	194	0.7039	0.8528	0.3089	0.7444	0.5364	0.9527
salicylaldehyde	35027	10360	42961	12248	30241	11272	32309	10283	30472	10892	32064	8631	0.2440	0.5076	0.3526	0.7837	0.8786	0.9575
ribose-5-phosphate	1020	389	646	184	1006	401	734	233	948	262	596	253	0.6675	0.8459	0.0028	0.0664	0.7804	0.9527
ribose	3127	716	3738	1077	2811	560	3002	714	2775	637	2690	685	0.1336	0.3880	0.4858	0.8338	0.6586	0.9527
ribonic acid	622	125	708	166	536	234	917	618	881	293	949	246	0.0902	0.3162	0.1052	0.4285	0.4673	0.9527
ribitol	1355	504	1781	558	1097	336	1349	589	1850	607	1681	840	0.1359	0.3890	0.5317	0.8338	0.4680	0.9527
pyrophosphate	15814	23029	16566	32371	46657	55416	9210	10043	8221	7785	4380	3235	0.4263	0.6462	0.1244	0.4681	0.8816	0.9575
putrescine	72196	27907	13666	3054	26726	10824	10405	6685	65646	22786	16935	6179	0.0002	0.0047	0.0000	0.0000	0.2113	0.9527
proline	8055	3847	7372	3581	4610	1251	5311	1289	7433	2145	8052	4014	0.1427	0.3951	0.9750	0.9838	0.8294	0.9527
pimelic acid	1416	717	1932	2405	1002	324	1047	200	866	340	1056	322	0.3164	0.5572	0.5928	0.8664	0.8197	0.9527
phosphoethanolamine	175028	75314	101396	100113	252932	104164	186096	89268	153121	72061	127727	85921	0.0667	0.2644	0.0334	0.2665	0.6435	0.9527
phosphate	1636812	696347	1489053	428650	1366850	617445	1827402	788176	1687401	424851	1437113	465784	0.9416	0.9706	0.9563	0.9767	0.5864	0.9527

phenylalanine	3213	1307	4433	2495	2139	319	1971	557	2265	401	2370	974	0.0469	0.2151	0.9014	0.9556	0.7191	0.9527
pantothenic acid	709	156	724	250	573	162	639	143	638	122	817	154	0.2426	0.5076	0.2224	0.6422	0.5443	0.9527
oxoproline	730958	132910	626212	151607	531248	129596	369325	95468	521280	115720	490749	210057	0.0041	0.0455	0.0375	0.2745	0.4994	0.9527
ornithine	47001	16828	72260	22926	27820	4340	33039	11304	37980	6384	33313	14801	0.0025	0.0310	0.4545	0.8243	0.1587	0.9527
oleamide	827	191	1021	449	741	219	911	82	985	164	765	195	0.7352	0.8611	0.6211	0.8670	0.0839	0.9527
N-acetylmannosamine	907	184	497	194	1173	535	844	321	699	217	503	177	0.0109	0.1048	0.0026	0.0664	0.5100	0.9527
myristic acid	8325	1795	7509	2295	9499	2920	9216	1313	9988	4195	10283	2553	0.1682	0.4361	0.8616	0.9452	0.7690	0.9527
myo-inositol	116469	22145	169615	39939	92799	12278	139044	65922	94770	11003	120906	29034	0.0449	0.2090	0.0021	0.0634	0.7977	0.9527
monomyristin	362837	88989	330779	83877	362181	73884	361407	56262	386444	99937	399979	55346	0.3869	0.6171	0.8691	0.9452	0.7561	0.9527
monoheptadecanoyl glyceride	24463	5197	22670	5457	23878	4707	24934	3702	26037	6155	26800	4208	0.4197	0.6391	0.9798	0.9857	0.7263	0.9527
methylmaleic acid	690	280	317	106	890	524	530	487	559	105	365	73	0.5729	0.7646	0.0053	0.0929	0.7786	0.9527
methionine	3727	1365	3786	1451	2403	609	2363	286	2509	479	3581	904	0.0150	0.1257	0.2911	0.7277	0.3140	0.9527
methanolphosphate	3609	1515	2644	1221	4314	1416	3268	1252	4479	2291	3488	500	0.2534	0.5084	0.0685	0.3642	0.8636	0.9548
mannose	2640	577	3539	555	2066	250	2718	962	2545	883	2081	308	0.0256	0.1618	0.2009	0.6215	0.1015	0.9527
mannitol	16221	17285	15237	15537	1813	906	2847	1022	10568	10903	11233	17078	0.0182	0.1436	0.8429	0.9429	0.6733	0.9527
maltotriose	321	90	589	432	261	32	358	83	253	74	323	78	0.0769	0.2926	0.0112	0.1505	0.7521	0.9527
malic acid	4328	2226	3300	829	11001	5421	7849	4393	3217	1961	2122	1180	0.0007	0.0122	0.1771	0.5993	0.8604	0.9544
lysine	1814	724	3280	891	713	147	853	382	1464	308	1273	621	0.0001	0.0021	0.4327	0.8243	0.0997	0.9527
levoglucosan	4026	6515	738	128	758	216	925	364	856	183	846	81	0.2500	0.5076	0.2059	0.6269	0.0603	0.9527
leucine	27009	5633	39917	8456	19280	3635	22563	6128	19300	5231	18800	12201	0.0055	0.0559	0.5184	0.8338	0.2709	0.9527
lauric acid	10154	2448	9385	2954	10586	3398	9290	1385	11022	4689	11790	2435	0.4315	0.6482	0.7597	0.9255	0.6186	0.9527
lactic acid	88004	58341	83446	41803	75933	20271	58393	20507	74653	35723	67041	20495	0.9626	0.9862	0.7707	0.9288	0.7102	0.9527
itaconic acid	13923	5011	6040	1115	114709	71429	19355	29608	7933	4020	5178	1707	0.0000	0.0004	0.0000	0.0022	0.0037	0.9527
isothreonic acid	7265	2486	4073	1621	7623	3704	4803	2047	6501	2308	4340	1545	0.8182	0.9001	0.0013	0.0555	0.8288	0.9527
isoleucine	71245	13724	99390	25633	41767	8197	53012	12432	53824	8443	54655	30056	0.0212	0.1441	0.6207	0.8670	0.3598	0.9527
inositol-4-monophosphate	3608	726	1820	1193	2863	1691	3586	1642	2984	1738	3083	1911	0.8382	0.9150	0.3912	0.7976	0.2204	0.9527
glycolic acid	3077	1090	3346	1921	2222	1079	4501	5064	3545	2137	3203	1162	0.7516	0.8695	0.4699	0.8324	0.6387	0.9527
glycine	39835	11659	37676	3585	21649	5495	31684	13368	25705	7444	33581	3886	0.0035	0.0424	0.0381	0.2745	0.2231	0.9527
glycerol-alpha-phosphate	2227	1155	1831	1323	3955	2247	3708	2585	2205	1094	2045	1020	0.0339	0.1742	0.4350	0.8243	0.9373	0.9807
glycerol-3-galactoside	850	167	519	189	651	255	735	197	882	215	609	134	0.6831	0.8497	0.0246	0.2340	0.0379	0.9527
glycerol	437007	144887	486952	71258	389457	126405	505342	101956	476084	111478	546969	96547	0.4548	0.6712	0.0647	0.3554	0.8152	0.9527
glyceric acid	4499	2810	4179	3054	2966	2141	7154	8727	6430	4823	5465	2746	0.4368	0.6533	0.5121	0.8338	0.3602	0.9527
glutamine	7160	4690	3424	2821	2486	847	2462	998	5184	2250	2609	1399	0.0441	0.2090	0.0084	0.1277	0.2018	0.9527
glutamic acid	21842	28870	14665	24038	26980	15807	9222	8681	17566	16164	15597	17479	0.7339	0.8611	0.0502	0.3002	0.8086	0.9527
glucose-6-phosphate	716	194	632	77	3602	2026	2641	1846	890	240	564	84	0.0000	0.0000	0.0861	0.4012	0.6966	0.9527
glucose-1-phosphate	4103	1356	3208	844	4147	2113	2849	1562	3101	1699	3334	1106	0.6631	0.8459	0.4400	0.8243	0.3559	0.9527
glucose	289968	88292	524601	150045	172552	28881	238693	58331	236984	68256	268582	79828	0.0001	0.0021	0.0018	0.0634	0.1639	0.9527

glucoheptulose	3050	871	1888	907	3466	1692	2676	1509	2746	1724	1008	514	0.0311	0.1710	0.0039	0.0766	0.3699	0.9527
fumaric acid	3737	1284	2484	925	7768	2962	4798	2004	2924	425	1902	446	0.0000	0.0004	0.0009	0.0420	0.9614	0.9940
fructose-6-phosphate	2534	766	639	128	3272	1733	1576	1181	2188	1286	650	132	0.0202	0.1440	0.0000	0.0000	0.4185	0.9527
fructose-1-phosphate	5221	1571	520	247	2195	1569	491	199	4562	2457	604	189	0.0271	0.1680	0.0000	0.0000	0.0805	0.9527
fructose	82855	22926	122963	22659	55258	7645	65344	21634	73451	12266	77642	16574	0.0002	0.0045	0.0327	0.2665	0.2164	0.9527
ethanol phosphate	979	126	629	278	823	211	791	104	961	370	822	174	0.6459	0.8370	0.0627	0.3502	0.1657	0.9527
erythritol	17225	3749	22295	3542	12258	1629	12912	2607	14572	1462	15461	2698	0.0001	0.0021	0.0797	0.3924	0.2943	0.9527
dodecanol	1857	243	1636	422	1731	678	1697	361	1810	468	1871	191	0.6859	0.8497	0.7798	0.9297	0.6013	0.9527
dihydrocholesterol	1185	364	1051	347	1337	788	1227	657	8466	16034	1144	664	0.5652	0.7605	0.2598	0.6937	0.4870	0.9527
diglycerol	10958	2575	9035	3126	13816	4243	8631	4956	11229	4811	15464	2948	0.3186	0.5572	0.3676	0.7855	0.0512	0.9527
deoxycholic acid	802	183	836	186	678	197	669	281	1101	499	813	415	0.3156	0.5572	0.5714	0.8546	0.7085	0.9527
dehydroabietic acid	601	169	1428	1337	793	136	761	193	973	370	846	139	0.7451	0.8666	0.2802	0.7190	0.1008	0.9527
cytidine-5-monophosphate	1285	573	1341	198	1332	408	961	441	972	348	901	142	0.1133	0.3451	0.4388	0.8243	0.3036	0.9527
cysteine	4083	4039	1368	509	1596	1171	1206	762	2227	990	2732	1250	0.0749	0.2883	0.2006	0.6215	0.3105	0.9527
creatinine	2147	1143	2997	3942	3729	3071	5333	6953	3968	3833	2377	714	0.2815	0.5285	0.7597	0.9255	0.8273	0.9527
conduritol-beta-expoxide	986	82	1416	333	1073	237	1178	532	881	157	1228	381	0.4910	0.7029	0.0217	0.2200	0.3798	0.9527
citrulline	1347	531	2266	967	1076	86	1416	211	1482	333	1288	335	0.1507	0.4104	0.0837	0.4007	0.0833	0.9527
citric acid	8020	6169	4507	5582	333530	179886	248676	166949	23669	3840	23758	10293	0.0000	0.0000	0.1182	0.4552	0.5635	0.9527
cholesterone	290	54	234	26	290	64	309	39	1040	652	795	277	0.0000	0.0001	0.5662	0.8529	0.6918	0.9527
cholesterol	512147	195581	432240	174992	694464	138056	594465	167897	616825	259261	638795	170136	0.0636	0.2567	0.4552	0.8243	0.6160	0.9527
beta-glycerolphosphate	950	74	773	145	1068	298	815	176	1007	181	829	171	0.7021	0.8528	0.0046	0.0858	0.9397	0.9807
beta-alanine	86946	38423	46927	27473	66275	25876	53238	21324	66736	23838	51192	18429	0.9530	0.9793	0.0106	0.1473	0.3734	0.9527
aspartic acid	36068	32282	10769	4388	55311	27075	31950	23113	19648	10667	18991	12888	0.0165	0.1345	0.0135	0.1614	0.2376	0.9527
arachidonic acid	3008	469	2806	714	3959	1134	3418	718	2747	600	2962	585	0.0671	0.2644	0.6356	0.8798	0.6207	0.9527
arabitol	2371	608	2768	927	2033	219	2030	726	2622	304	2664	466	0.0717	0.2792	0.7598	0.9255	0.7676	0.9527
alanine	21482	3882	22402	2982	15068	855	16068	2416	17898	3543	18976	3615	0.0003	0.0063	0.3556	0.7837	0.9975	0.9975
adenosine-5-monophosphate	3835	2190	2115	3226	7269	3763	4148	4274	3124	3325	2566	2713	0.0936	0.3162	0.0289	0.2618	0.6367	0.9527
adenosine	665	101	1157	1495	831	252	666	146	805	574	481	174	0.5102	0.7102	0.3646	0.7855	0.5840	0.9527
adenine	6633	3210	6560	4645	3330	1956	3633	3025	4464	2338	4304	4261	0.2070	0.4815	0.5402	0.8338	0.9370	0.9807
aconitic acid	262	36	201	74	599	384	434	207	216	109	231	52	0.0013	0.0176	0.4120	0.8118	0.5061	0.9527
5-aminovaleric acid	1606	472	957	461	691	376	971	492	1047	408	1044	769	0.1620	0.4284	0.5426	0.8338	0.1281	0.9527
4-hydroxybenzoate	623	87	540	65	658	362	570	169	991	976	679	135	0.4170	0.6378	0.4455	0.8243	0.9843	0.9965
4-aminobutyric acid	4336	1322	2865	643	4241	1466	3374	1058	4255	1186	3055	475	0.9073	0.9469	0.0078	0.1244	0.7986	0.9527
3-phosphoglycerate	1299	1430	246	14	564	388	328	96	421	181	506	418	0.9898	0.9979	0.1058	0.4285	0.3304	0.9527
3-hydroxypropionic acid	1969	852	2511	1276	1633	1177	3788	3951	2962	1737	2935	1480	0.5518	0.7484	0.1834	0.6114	0.4279	0.9527
3-Aminopiperidine-2,6-dione	9630	15242	102605	213974	11130	9889	2496	844	3333	1570	2060	744	0.0936	0.3162	0.7016	0.9242	0.0887	0.9527
3,6-anhydro-D-galactose	1391	396	2094	685	1180	381	1155	253	1277	59	1274	247	0.0142	0.1219	0.1857	0.6114	0.1412	0.9527

2-monopalmitin	392617	40094	354867	98342	444265	104449	418405	53454	397416	92846	484323	53156	0.1383	0.3914	0.8593	0.9452	0.1512	0.9527
2-ketoadipic acid	2261	415	1949	372	2113	819	2378	1170	1960	583	2289	450	0.9981	0.9981	0.7001	0.9242	0.4675	0.9527
2-hydroxyglutaric acid	891	492	670	318	1010	425	727	256	1014	420	677	116	0.7066	0.8528	0.0956	0.4207	0.9457	0.9831
2-deoxytetronic acid	1860	1458	1650	1133	1031	561	2728	3936	2495	2378	1911	1170	0.7302	0.8611	0.7035	0.9242	0.7346	0.9527
2,3-dihydroxypyridine	3024	2127	1876	1232	1471	449	2430	1199	2599	1510	1462	394	0.8195	0.9001	0.3796	0.7976	0.0722	0.9527
2,3-bisphosphoglyceric acid	7431	8094	5021	5748	1750	537	5091	8029	9714	13480	5150	8306	0.4849	0.7002	0.5364	0.8338	0.5277	0.9527
1-monopalmitin	2571217	481062	2646120	452621	2699658	423434	2640353	476053	2811226	462303	2967146	470667	0.3887	0.6171	0.7497	0.9255	0.8708	0.9575
1-hexadecanol	2459	1597	3053	2217	3457	2677	2711	1474	3107	1776	3272	1370	0.6471	0.8370	0.7970	0.9338	0.8475	0.9527
704730	5016	1105	5768	540	2645	524	3427	803	3945	521	4008	793	0.0000	0.0002	0.0555	0.3262	0.3614	0.9527
507164	6018	1795	2623	1419	4722	2528	5265	2227	4874	3072	6395	2378	0.8872	0.9465	0.9419	0.9703	0.1230	0.9527
505282	2619	1257	1688	1664	1843	1377	2615	1932	2386	1501	2956	2353	0.9120	0.9469	0.4156	0.8142	0.4896	0.9527
483122	2341	1382	2588	1851	1962	1811	4323	5905	2932	2494	2415	1516	0.9885	0.9979	0.6540	0.8977	0.6922	0.9527
474336	147	31	350	428	150	34	287	321	258	161	265	216	0.7102	0.8528	0.3140	0.7514	0.6828	0.9527
474043	634	194	5520	9849	668	184	599	83	703	212	2261	3630	0.3362	0.5776	0.0986	0.4236	0.2510	0.9527
471797	2997	418	2439	386	2607	821	3192	548	2589	430	3440	1108	0.7611	0.8695	0.3055	0.7416	0.0720	0.9527
425891	860	211	494	128	604	212	549	141	538	236	475	81	0.1744	0.4361	0.0409	0.2745	0.1391	0.9527
425774	1001	679	657	464	888	724	1563	462	1443	850	2201	1400	0.0516	0.2275	0.3961	0.7993	0.1698	0.9527
425770	3148	3478	1825	1970	4077	1603	2735	947	2720	2882	2551	1975	0.2063	0.4815	0.6312	0.8774	0.8434	0.9527
422802	2403	825	2315	1048	2087	614	2110	619	2129	591	1889	675	0.7645	0.8695	0.5753	0.8561	0.8874	0.9575
422263	1688	510	1240	228	1737	521	1561	553	1470	512	1954	495	0.5201	0.7111	0.7437	0.9255	0.0844	0.9527
421307	716	171	682	113	728	109	910	349	837	284	1099	441	0.1552	0.4193	0.2522	0.6937	0.5414	0.9527
421301	809	274	919	133	872	334	540	186	1096	420	1319	578	0.0311	0.1710	0.7328	0.9255	0.1739	0.9527
418155	5025	1069	4303	1444	6044	2158	4508	513	5303	1850	6242	592	0.2040	0.4813	0.4456	0.8243	0.1587	0.9527
413143	2039	634	1939	326	2197	858	2522	1328	2565	845	2240	483	0.4610	0.6744	0.9466	0.9703	0.7490	0.9527
412755	4266	798	3732	870	5770	1862	4463	775	4373	1731	4111	932	0.1344	0.3880	0.1763	0.5993	0.7328	0.9527
407455	1953	546	1818	1028	2163	927	2061	469	1564	526	1590	582	0.2494	0.5076	0.6929	0.9242	0.8441	0.9527
406766	5323	1083	6851	6243	4848	2272	3677	1079	4613	1991	3668	1840	0.2824	0.5285	0.3510	0.7837	0.8105	0.9527
402245	783	173	609	309	887	342	923	85	885	369	971	321	0.0923	0.3162	0.7314	0.9255	0.2890	0.9527
390227	2657	1180	4988	6761	1044	691	4565	7668	2308	3098	2553	1361	0.3114	0.5572	0.2679	0.7012	0.8828	0.9575
390218	1771	424	2704	773	1651	525	1916	283	1912	534	2136	618	0.2453	0.5076	0.0241	0.2340	0.4119	0.9527
390208	3686	1233	5788	2776	3639	984	3195	1023	3292	660	2644	1535	0.0798	0.2971	0.7736	0.9289	0.1538	0.9527
390194	1799	943	3082	1216	2100	810	1855	833	1317	639	2283	1954	0.4294	0.6479	0.1474	0.5217	0.2853	0.9527
390191	4385	1280	7030	1575	3825	1377	3897	1242	4512	1246	5172	995	0.0304	0.1710	0.0567	0.3275	0.2029	0.9527
390140	23474	8486	39479	12775	17503	4014	16680	4027	20268	3117	32663	8706	0.0009	0.0135	0.0074	0.1231	0.0732	0.9527
390137	878	296	974	444	624	346	992	851	983	552	1092	214	0.2611	0.5085	0.2391	0.6846	0.7858	0.9527
390126	1120	432	1571	403	813	165	984	233	1222	386	1445	408	0.0136	0.1199	0.0360	0.2745	0.7327	0.9527
390122	26347	7452	44465	8632	15691	3188	19756	6057	23627	6939	23319	8590	0.0001	0.0026	0.0313	0.2665	0.0976	0.9527

390121	97321	36885	69477	25213	83624	45462	47671	27141	73416	35830	73153	24004	0.2762	0.5276	0.1042	0.4285	0.2952	0.9527
390000	6248	3612	10270	7852	4680	2492	6350	4106	5488	3513	7558	4402	0.7604	0.8695	0.5223	0.8338	0.9920	0.9965
389332	15218	895	15199	2680	14305	2170	14629	1943	15640	5701	15630	1694	0.7085	0.8528	0.8006	0.9338	0.9479	0.9831
386902	2829	444	2270	767	4204	955	4065	964	1772	958	1942	760	0.0001	0.0021	0.7669	0.9274	0.4223	0.9527
385941	2037	788	3466	849	1718	522	1900	587	2173	546	2457	363	0.0214	0.1441	0.0187	0.1963	0.1677	0.9527
384894	2847	1005	32966	57280	3578	1542	3790	1139	2660	813	3709	652	0.0944	0.3162	0.0120	0.1541	0.0543	0.9527
382905	508	164	372	113	466	118	548	330	465	162	524	169	0.6770	0.8462	0.8197	0.9341	0.3609	0.9527
382070	960	379	819	186	1211	335	1417	465	1269	302	1050	233	0.0215	0.1441	0.6155	0.8664	0.4815	0.9527
381428	4423	1905	2715	1436	5250	2011	3790	1583	5267	2867	3750	1009	0.3680	0.6043	0.0394	0.2745	0.7609	0.9527
380488	1525	419	1327	419	1329	506	919	421	1038	173	1222	428	0.1727	0.4361	0.2870	0.7277	0.2808	0.9527
380390	482	211	438	250	879	913	875	698	586	398	2256	3662	0.2094	0.4815	0.3855	0.7976	0.4259	0.9527
379924	20314	5921	19018	5548	19533	8274	22119	3795	20814	4303	19061	3872	0.9776	0.9930	0.8488	0.9447	0.4915	0.9527
379691	1261	151	1414	801	954	285	1012	227	1159	335	1004	216	0.1121	0.3445	0.9222	0.9703	0.7193	0.9527
379611	1068	233	1631	714	927	201	1207	522	1050	366	1015	231	0.1736	0.4361	0.0862	0.4012	0.3833	0.9527
378571	1739	496	1327	419	977	309	1013	328	967	157	975	301	0.0055	0.0559	0.4225	0.8230	0.5285	0.9527
378468	21686	5616	27228	9174	14096	4911	18303	5928	16398	9186	23905	9174	0.2407	0.5076	0.1088	0.4338	0.7070	0.9527
377936	3843	1330	4462	746	3826	328	5783	3367	4514	1452	5607	1055	0.3929	0.6180	0.0433	0.2747	0.9183	0.9736
377932	4070	1289	3040	1735	5432	3290	5168	3387	4578	3743	3419	1798	0.4163	0.6378	0.3166	0.7523	0.8889	0.9575
377697	1591	727	1703	1108	1056	579	2653	2916	1805	1299	1702	681	0.9039	0.9469	0.2736	0.7105	0.4202	0.9527
377673	2451	477	2224	697	2986	1170	3549	1025	2941	627	2192	518	0.0531	0.2302	0.4526	0.8243	0.1677	0.9527
377430	4277	4502	7800	5415	11581	1769	7709	6548	12066	8361	8732	4693	0.2570	0.5085	0.5882	0.8642	0.2295	0.9527
376823	1361	377	1419	592	1244	371	1062	191	1220	336	1345	549	0.4509	0.6683	0.8886	0.9510	0.7637	0.9527
371546	325	139	642	701	509	328	391	249	446	274	717	517	0.6612	0.8459	0.4722	0.8324	0.6037	0.9527
371262	535	142	534	158	953	527	739	370	546	249	634	324	0.1287	0.3782	0.8444	0.9429	0.6396	0.9527
370761	4193	1009	4723	1174	4964	1359	5241	1050	5038	1659	5305	696	0.3662	0.6043	0.3440	0.7837	0.9853	0.9965
370223	3230	3558	3726	5276	8247	3786	5384	4006	4484	4067	4579	3535	0.0607	0.2534	0.6040	0.8664	0.7596	0.9527
369161	35322	32969	44280	10970	46203	8026	48922	8503	57147	16500	54405	10252	0.0966	0.3203	0.1869	0.6114	0.1923	0.9527
367101	4110	911	3308	803	4070	1099	4260	1086	4524	1708	5424	1884	0.1197	0.3613	0.9687	0.9818	0.2856	0.9527
366068	1300	235	1008	290	1197	574	1276	329	1618	378	1326	601	0.2607	0.5085	0.2630	0.6937	0.3086	0.9527
362282	884	234	632	260	820	569	919	243	765	182	610	180	0.7710	0.8695	0.5426	0.8338	0.1855	0.9527
360146	34175	7355	36878	9059	26522	6062	36994	22044	34534	12616	30398	6358	0.5028	0.7102	0.5251	0.8338	0.5191	0.9527
360128	572	597	359	135	291	121	242	127	518	651	329	230	0.3595	0.5992	0.4976	0.8338	0.9755	0.9965
360111	398	270	248	87	298	339	498	217	220	69	367	234	0.9128	0.9469	0.2138	0.6310	0.1176	0.9527
351520	8830	3069	9332	761	5928	893	5858	941	7010	1161	6328	1190	0.0008	0.0129	0.9701	0.9818	0.5396	0.9527
351518	1569	629	1002	236	1058	140	917	95	1124	284	847	129	0.0492	0.2227	0.0020	0.0634	0.3774	0.9527
351271	1303	312	6581	11581	1043	214	1126	362	1276	266	1127	249	0.1397	0.3914	0.3675	0.7855	0.3061	0.9527
351252	391	155	3260	5367	414	37	469	193	386	116	435	89	0.1015	0.3207	0.0383	0.2745	0.0655	0.9527

350283	494	137	451	122	381	183	685	695	560	367	524	215	0.8633	0.9274	0.5553	0.8456	0.5996	0.9527
349350	1121	552	2237	2660	1096	272	1153	346	1341	583	1046	225	0.8665	0.9274	0.6738	0.9138	0.4861	0.9527
347607	7821	2398	7667	573	8864	1577	9774	2050	9874	3812	7126	3396	0.3851	0.6171	0.5022	0.8338	0.2652	0.9527
346668	11007	24047	17605	25718	32419	30432	48325	28865	41814	42325	33566	30387	0.1676	0.4361	0.4550	0.8243	0.8115	0.9527
344959	2422	1510	1537	870	3045	1277	2233	274	2074	943	2184	1076	0.1817	0.4509	0.2438	0.6921	0.6305	0.9527
342919	1363	772	1149	119	1839	994	1648	464	1676	877	1422	588	0.4572	0.6718	0.9429	0.9703	0.9125	0.9704
341671	12670	4234	11261	3265	11257	4301	11031	3802	9267	3273	14046	4795	0.8991	0.9469	0.4748	0.8324	0.3061	0.9527
327608	315	178	379	91	454	73	472	82	470	166	421	147	0.0620	0.2534	0.5276	0.8338	0.4175	0.9527
327364	1269	1283	4115	4610	3122	2551	4081	2417	2469	2431	4265	3874	0.4958	0.7068	0.1480	0.5217	0.8077	0.9527
325583	2550	2255	3701	585	3320	753	3662	717	3249	2017	3905	206	0.4870	0.7002	0.0780	0.3900	0.4692	0.9527
247758	1382	606	1026	440	943	520	1141	351	1355	514	1372	320	0.2601	0.5085	0.9438	0.9703	0.3529	0.9527
247669	600	388	951	282	646	306	777	348	1009	638	833	532	0.8942	0.9469	0.5128	0.8338	0.2459	0.9527
241684	906	166	681	113	834	191	624	163	834	299	954	283	0.2452	0.5076	0.1400	0.5041	0.1031	0.9527
241681	581	83	675	121	550	143	739	280	696	362	903	268	0.3509	0.5923	0.0435	0.2747	0.8075	0.9527
241618	3668	989	2242	325	2439	561	1861	390	3365	1009	2277	350	0.0119	0.1077	0.0001	0.0071	0.5897	0.9527
241383	904	150	1077	236	969	259	1127	271	1056	526	1202	335	0.7448	0.8666	0.1106	0.4358	0.9927	0.9965
234849	1059	228	996	217	1127	221	1130	203	1154	316	1266	235	0.2500	0.5076	0.8410	0.9429	0.6768	0.9527
234848	25507	6099	24788	6036	26665	5827	27632	4288	28235	6796	30252	4996	0.2787	0.5276	0.7004	0.9242	0.8499	0.9527
234454	1310	339	1318	150	1486	438	1382	387	1616	754	1728	417	0.2928	0.5389	0.8169	0.9340	0.7836	0.9527
228119	1444	184	1425	583	1595	378	1358	366	1895	729	1658	299	0.2110	0.4815	0.2972	0.7321	0.8971	0.9602
224091	496	251	1075	433	399	99	529	164	513	244	469	221	0.1010	0.3207	0.1258	0.4684	0.1056	0.9527
223080	1194	279	1465	763	681	361	984	439	688	105	1056	344	0.0361	0.1780	0.0730	0.3811	0.6780	0.9527
210694	196285	54327	367649	70762	143604	37206	158638	42636	194365	37161	222662	50938	0.0001	0.0026	0.0038	0.0766	0.0374	0.9527
210313	1180	185	1250	215	934	220	846	258	976	337	1127	186	0.0255	0.1618	0.6817	0.9169	0.4430	0.9527
210272	5805	2124	8796	2506	3513	1102	4646	1365	4730	700	4677	2088	0.0038	0.0438	0.0967	0.4207	0.2301	0.9527
209677	968	991	603	179	1478	1102	926	1122	1109	1129	1818	1136	0.6911	0.8511	0.8874	0.9510	0.2397	0.9527
204704	2933	586	3093	792	1734	256	1983	561	1983	504	2628	503	0.0005	0.0086	0.0907	0.4135	0.4736	0.9527
172163	15931	5336	15207	4077	13039	1533	14017	1788	13894	4667	15378	4241	0.6716	0.8459	0.6084	0.8664	0.8118	0.9527
171968	7393	2708	11108	6369	7297	1117	8270	4004	7152	2218	5773	3020	0.2773	0.5276	0.8788	0.9510	0.2896	0.9527
171548	5208	2223	4794	1971	3589	635	4335	467	23223	40489	11340	16561	0.2113	0.4815	0.8095	0.9338	0.7709	0.9527
171286	13172	5811	20960	4253	8467	2004	11628	3579	13052	2449	11434	4270	0.0197	0.1436	0.0956	0.4207	0.0838	0.9527
170373	1904	629	1786	823	3618	2749	1343	370	1483	554	3101	3462	0.8957	0.9469	0.4622	0.8281	0.0602	0.9527
170303	1065	235	1529	2448	928	273	420	32	1035	400	3142	3723	0.2263	0.4923	0.3913	0.7976	0.3128	0.9527
170278	341	30	204	76	276	49	221	38	304	95	192	30	0.7192	0.8574	0.0001	0.0061	0.2871	0.9527
163829	800	295	1070	1617	981	1423	628	432	2189	2765	614	225	0.3788	0.6130	0.2468	0.6937	0.4457	0.9527
163410	407	101	3476	6735	386	66	324	59	389	78	491	153	0.2516	0.5077	0.2898	0.7277	0.3090	0.9527
145862	161531	24798	153638	44376	188583	48243	181959	31553	179296	34100	208096	20757	0.0796	0.2971	0.8306	0.9400	0.4448	0.9527

132103	424	141	433	172	313	102	482	113	507	162	276	79	0.7675	0.8695	0.6685	0.9104	0.0083	0.9527
131694	738	198	871	342	697	318	657	161	944	306	828	202	0.1603	0.4284	0.9128	0.9647	0.7116	0.9527
131101	4786	1027	8518	3770	5383	1790	4204	1071	5371	1699	6192	2405	0.2227	0.4923	0.3417	0.7837	0.0842	0.9527
128091	14211	3122	12714	3258	13723	2596	13750	2145	14527	3687	15228	2124	0.5011	0.7102	0.8288	0.9400	0.6213	0.9527
128024	2525	1012	2608	1095	2640	462	2948	1219	2902	459	3015	1173	0.6707	0.8459	0.9963	0.9974	0.9883	0.9965
126350	8544	2642	9248	2688	7723	2981	6715	2805	9136	1850	8407	1311	0.1466	0.4026	0.7225	0.9255	0.7162	0.9527
125982	4132	1495	2671	340	3343	1216	2092	648	3153	1241	5332	3349	0.0581	0.2466	0.3347	0.7752	0.0128	0.9527
125793	712	254	848	351	643	137	747	551	596	278	862	288	0.7675	0.8695	0.2157	0.6310	0.5493	0.9527
125472	496	206	582	162	612	278	795	320	643	385	679	211	0.5103	0.7102	0.1916	0.6114	0.9369	0.9807
121468	666	377	646	707	629	171	460	120	1036	913	529	285	0.7304	0.8611	0.0821	0.3988	0.8207	0.9527
121018	1206	341	1677	619	806	392	1166	357	1039	356	1116	406	0.0933	0.3162	0.0751	0.3814	0.5750	0.9527
119142	1239	779	1312	460	1290	1141	962	524	1312	424	912	367	0.7030	0.8528	0.5108	0.8338	0.4804	0.9527
119025	346	115	424	125	309	137	386	118	406	16	357	104	0.5965	0.7805	0.3766	0.7976	0.3040	0.9527
119023	2272	844	2665	1055	1515	771	1182	646	1840	433	1919	951	0.0195	0.1436	0.7204	0.9255	0.5639	0.9527
114258	6000	1834	4403	2599	3661	2246	4891	5225	6848	3743	5320	2170	0.2031	0.4813	0.5028	0.8338	0.6909	0.9527
113513	729	652	511	535	955	485	674	414	730	474	634	386	0.3194	0.5572	0.2022	0.6215	0.8728	0.9575
111253	7230	7395	14077	14375	1637	1231	2208	3144	1457	841	4332	5807	0.0299	0.1710	0.4623	0.8281	0.7760	0.9527
111058	1698	493	1699	583	1297	733	1606	408	1477	586	4459	7120	0.5962	0.7805	0.2626	0.6937	0.7013	0.9527
110702	823	366	759	62	561	104	511	203	684	165	563	135	0.0357	0.1780	0.3503	0.7837	0.7384	0.9527
110382	1840	1020	1682	856	4125	3470	1390	583	1194	380	2618	2838	0.4855	0.7002	0.4771	0.8324	0.0573	0.9527
110343	611	38	678	265	522	167	539	161	581	180	599	296	0.3518	0.5923	0.8167	0.9340	0.9733	0.9965
110289	647	114	539	69	495	127	539	215	588	89	859	789	0.3159	0.5572	0.9400	0.9703	0.5631	0.9527
110285	9282	2827	9014	723	5848	193	6574	1318	6596	1678	6328	1190	0.0009	0.0135	0.7199	0.9255	0.7908	0.9527
110265	3434	414	3175	636	3155	1053	2582	667	3605	1054	3474	328	0.1040	0.3256	0.2972	0.7321	0.6968	0.9527
110018	670	262	592	371	500	248	920	721	861	594	825	420	0.5842	0.7736	0.5385	0.8338	0.3392	0.9527
109387	3572	1582	2731	814	6205	1743	6290	1485	3421	1280	3269	1257	0.0009	0.0135	0.7150	0.9255	0.8969	0.9602
109386	1336	436	1165	231	916	161	1082	503	1235	305	1027	292	0.2160	0.4890	0.5677	0.8529	0.5212	0.9527
106742	1659830	656484	1522022	737555	990775	773310	1154279	569837	1173028	359079	907896	700534	0.2402	0.5076	0.6399	0.8821	0.4873	0.9527
106488	5533	1674	2656	1736	4130	1444	5760	4021	7210	5502	4034	1845	0.4130	0.6378	0.0584	0.3314	0.0879	0.9527
105366	15295	4360	14552	4155	18699	6437	15325	2480	17823	7026	19282	5577	0.2981	0.5433	0.7234	0.9255	0.6289	0.9527
105081	1054	409	915	138	926	229	1024	405	1294	532	1417	197	0.0444	0.2090	0.8029	0.9338	0.7552	0.9527
104394	700	142	549	210	1223	647	1163	366	699	353	670	207	0.0047	0.0510	0.5214	0.8338	0.6307	0.9527
104366	633	338	600	349	476	274	1011	1092	828	577	589	179	0.8638	0.9274	0.6842	0.9169	0.4096	0.9527
104365	1748	367	1908	293	2048	349	2627	1515	2196	650	1970	248	0.2495	0.5076	0.5416	0.8338	0.5646	0.9527
104248	2383	504	2091	596	2378	498	2373	380	2571	785	2669	348	0.2871	0.5314	0.7429	0.9255	0.5770	0.9527
103870	5243	1396	3382	982	4242	893	3922	400	4842	1574	4292	548	0.5794	0.7702	0.0251	0.2340	0.1329	0.9527
101742	4329	1280	3994	2141	2703	1326	5537	4583	5555	2685	4233	1317	0.4868	0.7002	0.5978	0.8664	0.1595	0.9527

100897	764	183	1157	372	749	252	780	141	942	321	897	519	0.4138	0.6378	0.3355	0.7752	0.3136	0.9527
100879	918	125	626	210	896	320	726	554	913	355	871	261	0.5618	0.7589	0.0739	0.3811	0.5076	0.9527
100032	40071	8700	36884	9424	38980	7338	40368	6425	42088	10660	43122	6752	0.5131	0.7102	0.9414	0.9703	0.7334	0.9527
100017	3740	527	2862	460	2590	1771	3060	1243	3843	955	3083	1022	0.2230	0.4923	0.7467	0.9255	0.2950	0.9527
97828	54158	29559	36136	18549	26883	19299	29122	9939	42003	29725	49299	17674	0.1724	0.4361	0.8545	0.9447	0.3961	0.9527
88501	747	140	714	272	616	206	662	132	910	379	798	279	0.1960	0.4791	0.7285	0.9255	0.6945	0.9527
84682	569	233	387	213	891	341	416	100	820	552	629	182	0.1402	0.3914	0.0156	0.1746	0.3581	0.9527
84181	5928	1075	5578	2252	6026	1238	5693	1143	6251	1806	6631	1183	0.5195	0.7111	0.7494	0.9255	0.6982	0.9527
84117	1623	418	1538	590	1786	267	1628	577	1874	782	2038	483	0.3421	0.5847	0.7898	0.9338	0.6197	0.9527
66431	504	229	503	205	489	236	492	233	379	190	661	164	0.9130	0.9469	0.2574	0.6937	0.3554	0.9527
51997	1194	320	1329	497	1372	490	854	398	1243	721	1127	384	0.6770	0.8462	0.4492	0.8243	0.3104	0.9527
48586	2506	1774	3052	2360	3144	2223	2688	1752	3267	2002	3550	1126	0.5170	0.7111	0.7636	0.9269	0.7702	0.9527
48468	3543	2969	5203	4813	4978	4762	4441	4203	4816	4426	4928	1265	0.7250	0.8611	0.5835	0.8610	0.7588	0.9527
47358	6912	1682	6902	1345	7105	2135	7571	1782	7064	1455	6764	1705	0.9269	0.9583	0.8836	0.9510	0.8457	0.9527
47242	426	174	337	91	303	152	330	67	441	171	364	109	0.2991	0.5433	0.6083	0.8664	0.4947	0.9527
46340	2466	1830	3741	3234	3350	3337	2802	2777	3270	2777	3290	1811	0.8000	0.8903	0.7141	0.9255	0.6707	0.9527
46292	3563	1973	2118	1241	4141	1193	3329	669	4199	1038	3834	1436	0.0324	0.1730	0.0487	0.2968	0.4455	0.9527
46231	1240	518	1786	1565	1542	1184	1518	1142	1677	1313	1316	590	0.9960	0.9981	0.9472	0.9703	0.7900	0.9527
46173	3743	2396	5268	1245	2788	1791	5792	943	3679	2263	6300	1676	0.8094	0.8979	0.0023	0.0639	0.7707	0.9527
45353	1608	131	1077	209	1397	298	1322	387	1820	725	2011	429	0.0067	0.0661	0.2096	0.6310	0.0631	0.9527
43100	4820	2497	4070	4326	4829	2671	2615	1262	7007	5310	2560	1148	0.8518	0.9227	0.0133	0.1614	0.7061	0.9527
42445	670	224	689	201	560	302	629	197	484	170	436	158	0.0980	0.3207	0.7430	0.9255	0.6967	0.9527
42424	781	448	504	101	902	495	648	534	598	250	475	191	0.5066	0.7102	0.1062	0.4285	0.8475	0.9527
42357	1980	367	1669	541	1847	714	2114	1100	2500	1373	1968	510	0.5952	0.7805	0.5177	0.8338	0.5964	0.9527
41985	1249	357	1165	590	1251	379	873	360	1092	549	1064	308	0.8126	0.8984	0.3615	0.7855	0.5344	0.9527
34169	1422	1209	1517	1169	774	538	2169	2748	2337	2284	1526	1089	0.7807	0.8747	0.5054	0.8338	0.5433	0.9527
34116	68479	37641	68858	33352	26025	15225	22536	18770	54814	19417	45735	34606	0.0337	0.1742	0.3812	0.7976	0.6955	0.9527
34097	1097	221	6331	11711	917	259	1005	176	1097	437	798	239	0.1913	0.4712	0.5403	0.8338	0.2992	0.9527
33993	1783	505	1183	716	1450	1546	1913	1927	3261	2431	1381	467	0.3470	0.5900	0.2123	0.6310	0.2033	0.9527
33468	3020	1060	2991	1678	2416	882	3386	522	3001	1048	3366	661	0.7734	0.8695	0.2812	0.7190	0.3880	0.9527
31563	668	166	841	648	520	118	575	124	632	298	500	160	0.3000	0.5433	0.9262	0.9703	0.6076	0.9527
31459	16404	7278	10920	4466	12462	4084	10449	2474	15422	6037	11302	6689	0.9685	0.9892	0.1229	0.4680	0.8320	0.9527
31448	4009	830	3295	1281	4223	1754	3563	906	4833	2546	6086	2825	0.0882	0.3162	0.7796	0.9297	0.3027	0.9527
31408	6007	943	4788	1226	4702	1476	5426	1238	5980	2596	6177	1226	0.3551	0.5947	0.9693	0.9818	0.2356	0.9527
31362	2750	495	2356	699	2232	901	2959	1403	2316	1176	2486	660	0.8493	0.9227	0.6018	0.8664	0.4628	0.9527
31223	135843	85249	74786	81186	179951	75915	123920	70815	145180	120480	100321	59302	0.1624	0.4284	0.0299	0.2640	0.6422	0.9527
29926	1955	752	1642	725	2286	1234	3158	1145	2594	1814	2799	1545	0.2237	0.4923	0.5667	0.8529	0.4396	0.9527

29923	1321	294	1531	360	1170	298	1324	214	1425	225	1495	245	0.1985	0.4813	0.1675	0.5786	0.8561	0.9528
26736	1200	909	896	804	507	128	624	483	856	1093	729	499	0.5713	0.7646	0.8523	0.9447	0.7650	0.9527
21622	4840	775	4346	747	4584	836	4776	572	4871	1327	4911	646	0.7640	0.8695	0.8659	0.9452	0.5664	0.9527
21511	2518	667	2500	416	2586	868	2798	443	2913	1282	3149	524	0.3782	0.6130	0.3928	0.7976	0.8832	0.9575
18263	10421	7670	9430	8053	4681	3862	15888	23569	12588	13708	10790	7626	0.7954	0.8882	0.5221	0.8338	0.4624	0.9527
17288	1820	1428	1911	1550	793	618	3105	4328	2041	1819	1667	1174	0.8538	0.9227	0.3869	0.7976	0.3459	0.9527
17225	1007	195	809	309	719	168	637	222	731	123	704	155	0.0425	0.2063	0.1161	0.4524	0.6503	0.9527
16809	723	210	442	116	558	207	408	238	609	147	463	142	0.3314	0.5753	0.0140	0.1623	0.8531	0.9527
16788	550	238	590	178	425	84	361	126	548	120	474	125	0.0536	0.2302	0.4679	0.8324	0.5275	0.9527
16747	815	125	1096	277	663	153	1250	794	731	173	725	198	0.2029	0.4813	0.0415	0.2745	0.2071	0.9527
16561	4054	422	7530	6739	2254	1205	4069	1683	3143	1308	3609	867	0.0502	0.2242	0.0333	0.2665	0.5117	0.9527
13146	664	409	475	235	890	552	701	389	537	233	408	142	0.1004	0.3207	0.1495	0.5217	0.9935	0.9965
12444	467	199	472	198	283	109	496	421	553	364	418	105	0.3780	0.6130	0.5408	0.8338	0.5119	0.9527
10176	3740	527	3018	407	4085	561	2764	1181	3843	955	3116	755	0.9105	0.9469	0.0033	0.0739	0.4588	0.9527
7490	3296	2640	1216	361	2077	1293	1314	566	3396	1598	2992	1449	0.0247	0.1618	0.0460	0.2856	0.3876	0.9527
7458	1274	307	1262	584	1597	915	1257	428	1312	540	1497	1120	0.9781	0.9930	0.7920	0.9338	0.9736	0.9965
6646	1354	462	1729	1098	923	358	648	212	692	268	2155	1053	0.0301	0.1710	0.1276	0.4697	0.0096	0.9527
5990	4277	1801	4713	1248	5367	1410	5602	1510	5144	1200	4765	1434	0.2656	0.5143	0.8037	0.9338	0.7044	0.9527
5388	94881	41524	54540	19698	88462	46807	53840	33620	80567	44928	92896	23929	0.3886	0.6171	0.1906	0.6114	0.3113	0.9527
5259	1525	371	1210	341	1572	498	1349	300	1840	546	1729	361	0.0988	0.3207	0.2166	0.6310	0.7474	0.9527
5245	33667	25226	28126	25623	27764	16923	21656	20420	37841	45637	37835	27226	0.9920	0.9979	0.5238	0.8338	0.4437	0.9527
5244	13377	5318	10687	4729	14650	9588	11532	4188	15592	13485	15982	5125	0.6644	0.8459	0.7556	0.9255	0.5470	0.9527
4983	3670	798	3439	1800	2682	614	3175	1973	3620	1059	3061	488	0.3913	0.6180	0.5775	0.8561	0.7286	0.9527
4929	1706	304	14329	25160	2362	388	2448	419	1474	516	1419	350	0.0343	0.1742	0.1013	0.4285	0.0919	0.9527
4793	1496	675	1571	566	775	462	778	267	1174	157	1113	340	0.0325	0.1730	0.5497	0.8408	0.7270	0.9527
4745	662	119	1144	724	574	143	1108	1003	499	174	596	191	0.1109	0.3441	0.0417	0.2745	0.7704	0.9527
4716	1156	185	811	98	1258	384	1061	317	915	111	985	200	0.2262	0.4923	0.0913	0.4135	0.1509	0.9527
4609	2664	995	3647	940	1847	235	2616	821	2520	504	2966	698	0.0276	0.1680	0.0098	0.1422	0.6968	0.9527
4534	686	69	688	102	609	76	715	136	564	198	714	129	0.5117	0.7102	0.0666	0.3600	0.3273	0.9527
4361	4787	665	4305	2087	3766	2112	5329	770	6420	2393	5307	1664	0.2596	0.5085	0.7908	0.9338	0.1306	0.9527
3423	1542	403	1720	482	1844	660	1879	319	2125	622	2033	490	0.1740	0.4361	0.6565	0.8977	0.8532	0.9527
3206	3155	1348	4028	2127	3444	855	4302	819	4008	1323	4529	935	0.3359	0.5776	0.1374	0.5001	0.9351	0.9807
2847	2933	586	2928	565	1436	397	1622	828	1964	287	2069	709	0.0001	0.0021	0.8111	0.9338	0.9789	0.9965
2551	1098	271	915	356	1114	239	884	281	986	298	1191	244	0.7178	0.8574	0.4282	0.8243	0.1797	0.9527
2475	7679	2681	7448	2124	9867	2973	9044	2045	9129	2088	10195	1639	0.0816	0.3003	0.8969	0.9538	0.7641	0.9527
2469	7880	2183	9047	2391	8707	3152	9961	2100	9303	4232	7654	4009	0.6136	0.7998	0.8879	0.9510	0.4090	0.9527
1981	3555	855	2491	1081	1967	605	2708	2645	3986	2927	3288	733	0.1256	0.3725	0.6150	0.8664	0.5043	0.9527

1725	492558	404812	231904	368967	617715	684392	345552	337386	568018	513955	660148	610066	0.4091	0.6374	0.4006	0.8034	0.3347	0.9527
1702	5510	1079	7824	7829	3840	778	3902	1345	4021	1266	3376	1230	0.0613	0.2534	0.7478	0.9255	0.8337	0.9527
1686	336800	65410	263710	61879	381409	134081	294495	44869	376771	224783	407160	94977	0.2241	0.4923	0.3245	0.7602	0.2281	0.9527
1684	345	106	207	70	219	111	269	157	251	188	248	95	0.6605	0.8459	0.9305	0.9703	0.3808	0.9527
307	10711	3442	15431	3721	6742	1869	9372	3036	8621	1130	7173	2862	0.0014	0.0193	0.1960	0.6194	0.0600	0.9527
228	2334	652	3348	2713	2071	553	1677	339	1996	607	2885	763	0.2012	0.4813	0.4029	0.8034	0.2507	0.9527
134	3351	1626	5865	3612	4318	2410	3218	1906	3786	1912	3196	1501	0.6873	0.8497	0.8940	0.9538	0.2886	0.9527
98	59835	24650	44755	24070	73490	29891	61506	21566	77993	40161	68259	23791	0.1238	0.3703	0.1886	0.6114	0.7815	0.9527
